# The origins of evil: From lesions to the functional architecture of the antisocial brain

**DOI:** 10.3389/fpsyt.2022.969206

**Published:** 2022-10-25

**Authors:** Jules R. Dugré, Stéphane Potvin

**Affiliations:** ^1^Research Center of the Institut Universitaire en Santé Mentale de Montréal, Montreal, QC, Canada; ^2^Department of Psychiatry and Addictology, Faculty of Medicine, University of Montreal, Montreal, QC, Canada

**Keywords:** antisocial behaviors, brain lesions, meta-analytic connectivity modeling, neuroimaging, co-activation

## Abstract

In the past decades, a growing body of evidence has suggested that some individuals may exhibit antisocial behaviors following brain lesions. Recently, some authors have shown that lesions underpinning antisocial behaviors may disrupt a particular brain network during resting-state. However, it remains unknown whether these brain lesions may alter specific mental processes during tasks. Therefore, we conducted meta-analytic co-activation analyses on lesion masks of 17 individuals who acquired antisocial behaviors following their brain lesions. Each lesion mask was used as a seed of interest to examine their aberrant co-activation network using a database of 143 whole-brain neuroimaging studies on antisocial behaviors (*n* = 5,913 subjects). We aimed to map the lesion brain network that shows deficient activity in antisocial population against a null distribution derived from 655 control lesions. We further characterized the lesion-based meta-analytic network using term-based decoding (Neurosynth) as well as receptor/transporter density maps (JuSpace). We found that the lesion meta-analytic network included the amygdala, orbitofrontal cortex, ventro- and dorso-medial prefrontal cortex, fusiform face area, and supplementary motor area (SMA), which correlated mainly with emotional face processing and serotoninergic system (5-HT_1A_ and 5-HTT). We also investigated the heterogeneity in co-activation networks through data-driven methods and found that lesions could be grouped in four main networks, encompassing emotional face processing, general emotion processing, and reward processing. Our study shows that the heterogeneous brain lesions underpinning antisocial behaviors may disrupt specific mental processes, which further increases the risk for distinct antisocial symptoms. It also highlights the importance and complexity of studying brain lesions in relationship with antisocial behaviors.

## Introduction

Antisocial behaviors are generally defined as a behavioral manifestation of serious disregard for and violation of others’ rights such as property destruction, theft/robbery, and aggression (e.g., physical and sexual aggression) (1). In the past decades, a growing body of evidence has linked traumatic brain injuries (2, 3), repeated concussions [e.g., Chronic traumatic Encephalopathy: (4, 5)] and resection of brain tumors (6–8) to the emergence of antisocial behaviors and aggressive tendencies including theft/robbery, destruction of property, physical assault, and murders. Indeed, it has been shown that sport-related concussions may increase the risk for impulsivity and aggressive behaviors (9) which is potentially due to sport-related impacts on frontal and temporal areas (10). These results indicate that medial prefrontal cortex (mPFC) and anterior temporal lobe (ATL) may be particularly relevant for the emergence of antisocial behaviors. For instance, in a recent meta-analysis of task-based functional neuroimaging studies, we found that individuals with antisocial behaviors exhibited deficient activity of the mPFC during social cognition tasks, whereas amygdala activity negatively correlated with the severity of antisocial behaviors across neurocognitive domains (11). Through a meta-analysis of resting-state connectivity studies in antisocial population, we also showed evidence that both amygdala and mPFC (i.e., ventral and dorsal) exhibited disrupted connectivity (12). Moreover, there is a growing body of evidence suggesting the importance of serotoninergic [mainly serotonin transporter (5-HTT) and 5-HT_2A_ and 5-HT_2B_ receptors] and dopaminergic systems [mainly Dopamine transporter (DAT) and D_2_ and D_4_ receptors] in our comprehension of antisocial behaviors (13–16). Indeed, these systems are known to encompass the amygdala/ATL and mPFC (e.g., mesolimbic/mesocortical dopaminergic pathways) (17, 18). Although such convergent results underscore the importance of the amygdala/ATL and mPFC, the neurobiological pathways linking brain lesions to antisocial behaviors remain largely unknown. Furthermore, characterizing these neurobiological pathways using receptor/transporter density maps (19) may inform us about the potential treatments for reducing antisocial behaviors following brain lesions.

Most of the current knowledge about neurobiological markers of antisocial behaviors relies on correlative methods. Lesion studies are therefore crucial as they offer a more causal association between the lesion and the emergence of symptoms. Recently, Darby et al. (20) examined the common brain network across 17 lesion cases that were temporally associated with aggression and antisocial behaviors (i.e., using lesion-to-voxel analysis on *n* = 1,000 healthy subjects during resting-state). The authors observed that the lesions were positively connected with brain regions implicated in the DMN but negatively connected with brain regions spanning the medial visual (lingual/calcarine), ventral (aINS, dACC/aMCC, and pre-SMA) and dorsal attention (SPL, FEF) networks during resting-state. Moreover, they showed that the lesion network linked to antisocial behaviors was functionally characterized by moral decision-making, in comparison to the network of control lesions (i.e., associated with other syndromes) (20). However, the lesion network mapping employing resting-state data in healthy subjects does have several limitations. First, using resting-state data limits our ability to understand what mental processes are specifically disrupted by the lesion. Indeed, a lesion to a particular region (e.g., amygdala) may exhibit more pronounced symptoms (e.g., aggressive behaviors) when faced with a particular context (e.g., threatening stimulus) compared to another (e.g., language processing). Despite that Darby et al. (20) found that the lesioned network was associated with moral decision making, antisocial subjects show prominent neural dysfunctions during fMRI tasks involving threat detection and cognitive control (11). The use of task-based fMRI studies is therefore of utmost importance as it offers the possibility to examine the heterogeneity of mental processes disrupted by lesions. A second limitation of the lesion network mapping using healthy subjects is that it remains unknown whether lesions lead to reorganization and/or compensation of neural processes which may be responsible for the emergence of a symptom (21). If this holds true, the neural reorganization and/or compensation should closely resemble the functional architecture observed in subjects exhibiting the same symptom. Therefore, using data from subjects exhibiting antisocial behaviors would enhance our ability to map brain co-activation (between the lesion and whole-brain voxels) that are specifically associated with antisocial behaviors.

The current study aims to overcome these limitations to better characterize the lesion-based networks associated with the emergence of antisocial behaviors. In our study, the lesion network mapping was conducted through meta-analytic connectivity modeling (MACM) using 143 whole-brain fMRI studies comprising 5,913 subjects exhibiting antisocial behaviors (i.e., conduct problems to conduct disorder and antisocial behaviors to antisocial personality disorder). We examined the task-based lesion network at a group level (across the 17 lesions) compared to 655 control lesions and identified its corresponding mental function and associated receptor/transporter density maps. Moreover, we investigated whether specific antisocial behaviors may increase the heterogeneity across lesions and conducted additional analyses to group homogeneous lesion-based networks and identify reliable lesion brain networks associated with antisocial behaviors.

## Materials and methods

### Antisocial and control lesions

**FIGURE 1:**
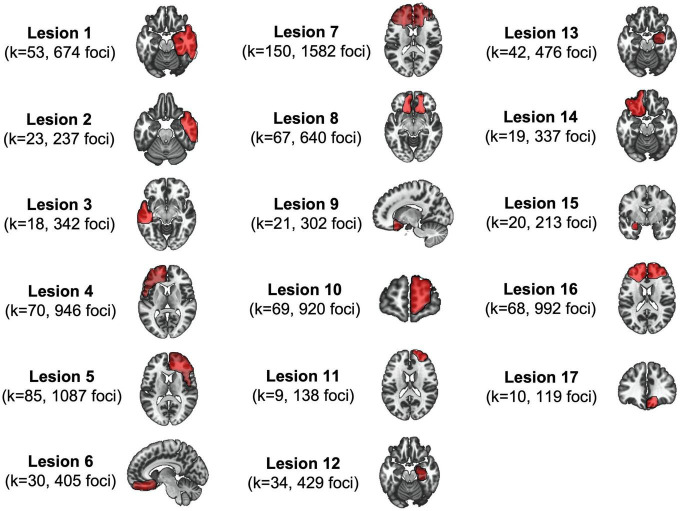
The 17 lesions associated with antisocial behaviors. This figure shows lesion masks (seeds) as well as their respective meta-analytic informations derived from the Antisocial Brain DataBase.

Lesioned patients were identified through a literature review. Inclusion criteria included: (1) case description of antisocial behaviors; (2) a brain lesion; (3) a published image of the brain lesion of adequate quality to conduct manual tracing onto a standardized template (MNI space). Darby et al. (20) retrieved 40 cases but 17 had explicitly described that the lesion preceded the antisocial behaviors. In the current study, we only used these 17 binary masks: (see Figure 1). More detailed information about the method and sample can be found elsewhere (20). The mean age at lesion was 23.56 years old (SD = 17.27) and antisocial behaviors were subsequently reported, on average, 9.09 years later (SD = 11.20). Lesions mainly included tumors (*n* = 6) and traumas (*n* = 6). Antisocial behaviors included murder, sexual and physical aggression, illegal financial decisions, theft/robbery, and destruction of property. Based on each of the lesioned patient’s case study, we manually coded their antisocial symptoms, following the Structured Clinical Interview for DSM-IV-TR [SCID-II; (22)]. Symptoms included: deceitfulness (i.e., repeated lying, use of aliases, conning others for personal profit), irritability/aggressivity (i.e., initiation of physical fights, assaults), irresponsibility (i.e., failure to sustain consistent work behavior, failure to honor monetary obligations), and limited prosocial emotions (LPE) (i.e., lack of remorse or guilt, callous-unemotional, or lack of empathy, deficient affect). For each symptom, we coded the absence (0) or presence (1) if the patients exhibited *at least* one of the associated behaviors. Across the 17 cases, 5 lesioned patients exhibited deceitfulness, 12 behaved aggressively, 9 showed irresponsibility, and 10 reported LPE.

Control lesions were also used to extract lesion network that is specific to antisocial behaviors. The control lesions were 655 manually segmented lesion masks from the Anatomical Tracings of Lesions After Stroke (ATLAS) dataset (23), which were gathered from 44 research cohorts across 11 countries involved in the ENIGMA Stroke Recovery working group (24). Lesions were mainly distributed in subcortical regions but also spanned cortical (25.5%) and other areas such as the brainstem (14.8%).

### Antisocial brain database

Meta-analytic connectivity modeling [i.e., MACM; (25–27)] is often used to examine which brain regions are co-activated with a defined seed region (i.e., lesion mask) in healthy subjects. This approach identifies all experiments that reported at least one peak coordinate within a seed region and meta-analyses them via a coordinate-based algorithm [e.g., activation likelihood estimation (ALE)]. Convergent results would thus indicate significant co-activated brain regions. However, given that we aimed to examine the altered co-activation in antisocial subjects here we used data from the Antisocial Brain Database,^1^ an initiative to collect neuroimaging data (similarly to the BrainMap database) to better characterize brain dysfunctions in subjects exhibiting antisocial behaviors. The database comprises 143 original studies (5,913 subjects) which included a total of 323 contrasts of aberrant co-activation observed in subjects exhibiting antisocial behaviors (across hyper- and hypoactivation) (see Supplementary material for the complete list of studies). Experiments included subjects that were assessed for antisocial behaviors or conduct problems or were diagnosed with a conduct disorder or antisocial personality disorder. Most experiments focused on negative stimuli (48.3%), social cognition (33.7%), positive stimuli (18.3%), and cognitive control (14.2%). Studies were retrieved from recent systematic review and meta-analyses on task-based activation (11, 12, 28–37). Articles were included if they met the following criteria: (1) original paper from a peer-reviewed journal, (2) using a sample without any comorbid major mental illness or organic impairment, and (3) employed a voxelwise (whole-brain) case-control and/or dimensional analysis.

### Meta-analytic connectivity modeling

Here, we performed MACM using the specific co-activation likelihood estimation (SCALE) algorithm to extract spatially convergent peaks coactivating with each of the 17 lesion masks with the Neuroimaging Meta-Analysis Research Environment package for python [NiMARE; (38)]. The standard MACM procedure includes extracting experiments that reported activation in a particular seed region, then conducting a meta-analysis using the revised version of the ALE algorithm (39). For each experiment, a modeled activation (MA) map is created by modeling coordinate foci with a spherical Gaussian probability distribution, weighted by the number of subjects to account for spatial uncertainty due to template and between-subject variance (40). It also ensures that multiple coordinates from a single experiment do not jointly influence the MA value of a single voxel. Voxel-wise ALE scores were then computed as the union of MA maps, which provide a quantitative assessment of convergence between brain activation across experiments. However, one limitation of this standard approach is that the base rate of activated voxels, in the whole database, may bias results when running standard MACM on the selected experiments. Therefore, Langner et al. (41) developed a new method that considers the *a priori* probability of finding activation across voxels, namely the SCALE. A voxel-specific null distribution is thus computed through a Monte-Carlo simulation which included shuffling coordinates from the original database. After 10,000 iterations, a null distribution of expected convergence is generated given the base rate in the database. In our study, we extracted studies of individuals with antisocial behaviors that reported at least one peak coordinate within each lesion mask. Moreover, rather than computing a null distribution from the Antisocial Brain Database, we rather extracted the coordinates underlying control lesions MACM maps to compute the null distribution. This yields lesion-MACM maps that are specific to antisocial behaviors in comparison to control lesions. Voxelwise z-score map was then extracted for each of the 17 lesion-specific MACM maps. Additionally, usual lesion network mapping utilizes statistical thresholding ranging from *p* < 0.05 uncorrected (42) to pFWE < 10^–11^ (43), using a one-sample *t*-test in healthy subjects. Given the complexity of our analyses [i.e., (1) using database of aberrant brain activity (compared to normal functioning); (2) using coordinates of control lesions (as opposed to the base rate of activations) to compute the null-distribution] we decided to use a more lenient threshold (*p* < 0.05 uncorrected). After the thresholding, the voxelwise z-score maps were binarized and summed to examine the overlap between lesions at a group level, namely, the task-based lesion network. Additionally, we examined how strongly the co-activation observed in antisocial subjects deviates from normal functioning by comparing lesion-specific MACM map in antisocial subjects with their respective map in healthy subjects (BrainMap database).

### Investigating the heterogeneity in co-activation networks

Given that results at a group level may be driven by some lesions, we conducted additional analyses to examine the heterogeneity between lesion networks. First, we aimed to unveil whether antisocial symptoms showed distinct associations with Neurosynth terms. To do so, we computed spatial similarity between the 17 lesion-based MACM maps and Neurosynth meta-analytic terms such as fear, faces, reward, and gain. Then, through point-biserial correlation, we examined whether the spatial similarity coefficient was associated with the presence of specific antisocial symptoms (i.e., Deceitfulness, Irresponsibility, irritability/aggressivity, and LPE symptoms). This allowed us to examine whether MACM resembles a term neural map, the stronger or weaker the association with specific antisocial behaviors.

**FIGURE 2:**
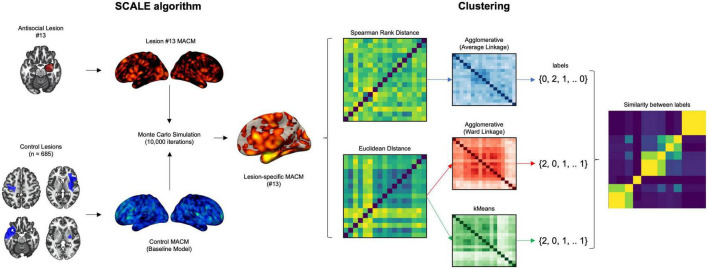
Workflow of the analyses to examine the heterogeneity in lesion network associated with antisocial behaviors. Investigating disentangling the heterogeneity of meta-analytic networks across lesion underpinning antisocial behaviors. Experiments that reported activation foci within each of the antisocial lesion were identified and compared 10,000 times to a null distribution of control lesion (stroke dataset). Pairwise distances (Spearman Rank and Euclidean) were computed between the resulting voxelwise z-score maps. Agglomerative and kMeans clustering methods were run to extract homogenous subgroups of networks.

To examine the neural heterogeneity across MACM maps (please refer to Figure 2 for the workflow), we first computed the distance between lesions. To do so, we converted the lesion images (Voxelwise z-score maps) into 17 one-dimensional vectors representing subjects by voxels (17 lesions by 902,629 voxels). Then, we computed pairwise Euclidean distance between subjects as well as Spearman Rank Correlation distance (1 – *r*) and ran 3 different clustering algorithms: (1) Agglomerative clustering using the Spearman Rank correlation distance and average linkage, (2) Agglomerative clustering using Euclidean distance and Ward linkage, and (3) kMeans using Euclidean distance. To extract the most optimal number of clusters, we examined silhouette, calinski-harabasz, adjusted rand index as well as variation of information for 2–5 cluster solutions, as done recently (44). When the most optimal number of clusters was found, we summed lesions MACM maps that defined each cluster to identify convergent brain regions. Finally, we applied dendrogram on the (dis)-similarity between all clusters of the three algorithms for interpretability purposes.

### Functional characterization

We functionally characterized the MACM networks using Neurosynth term-based decoding [i.e., only the top 10 terms; (45)] as well as whole-brain receptor/transporter density maps (19) (see list in Supplementary material). Briefly, we assessed similarity (Spearman correlation) with 28 receptor/transporter density maps distributed across 8 neurotransmitter systems including dopamine, noradrenaline, serotonin, acetylcholine, glutamate, GABA, cannabinoid, and opioid (see JuSpace v1.4).^2^ Exact permutation-based *p*-values (with 1,000 permutations) were computed and then corrected using false discovery rate (FDR) for the number of tests.

## Results

### Lesion-based meta-analytic co-activation modeling

To examine whether lesions may lead to reorganization and/or compensation in neural processes underlying antisocial behaviors, we assess the spatial similarity between lesion-specific co-activation in antisocial subjects and their normal co-activation (BrainMap). Results indicated small spatial similarity between lesion-specific co-activation in antisocial and healthy subjects (mean *r* = 0.21, S.D = 0.06), the correlation strength between antisocial and normal functions ranged from *r* = 0.07 (#7) to *r* = 0.29 (#2).

**FIGURE 3:**
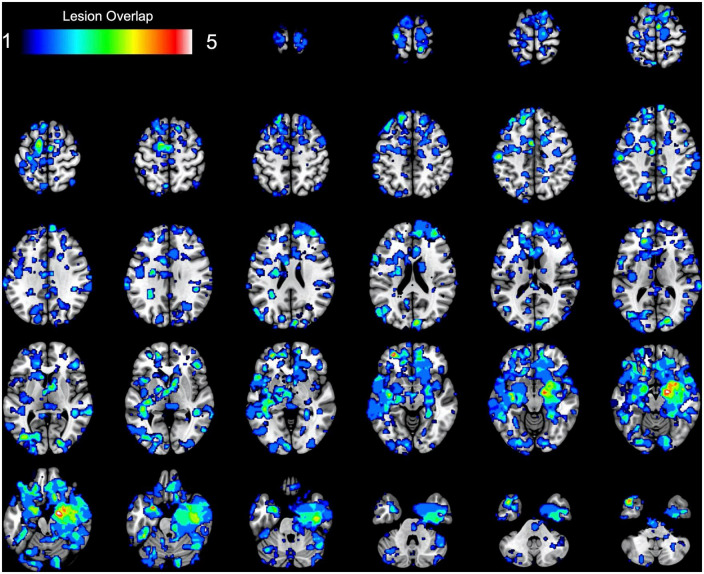
Results of the task-based lesion network mapping. The figure represents the spatial overlap between binarized lesion-based meta-analytic maps.

At a group level, overlapping the 17 MACM maps (thresholded and binarized) revealed deficient task-based co-activation between bilateral amygdala, medial and lateral OFC, SMA, ventro- and dorso-medial PFC, fusiform area, and visual V4 area (see Table 1 and Figure 3). However, the overlap between lesion-based meta-analytic maps was only weak (∩ ≤ 5 out of 17 maps, ≈ 29.41%).

**TABLE 1:** Results from the lesion-based meta-analytic connectivity modeling.

Regions	MNI coordinates			Lesion contribution (%)
	x	y	z	
				
AMY	18	–8	–22	29.41
AMY	–26	2	–26	29.41
mOFC	–18	20	–16	29.41
lOFC	–32	50	–8	29.41
SMA	8	–4	62	29.41
Fusiform face area	–38	–42	–24	23.53
vmPFC	14	54	–4	23.53
dmPFC	–14	42	44	23.53
aHIP	–34	–18	–12	23.53
hOc4la	–38	–78	4	23.53
Temporal pole	–42	10	–42	23.53

The overlap between task-based MACM images was performed by overlapping thresholded and binarized images. Only regions showing equal or more than four peaks are reported.

Functional characterization using Neurosynth revealed that the overlap between lesion-based MACM maps was mainly associated with emotional (i.e., neutral, emotional, fear, fearful, happy) and facial terms (e.g., neutral faces, expression, facial) with coefficients *r* > 0.24. Also, the task-based lesion network spatially correlated with 5-HT_1A_ (*r* = 0.34, [^11^C] CUMI-101 radioligand) as well as serotonin transporters 5-HTT (*rs* = 0.27–38, [^11^C]MADAM and [^11^C]DASB radioligands), after applying the FDR correction.

### Disentangling the clinical heterogeneity

We carried out subsequent analyses to investigate whether spatial similarity between lesion-specific meta-analytic networks and Neurosynth meta-analytic terms may be associated with distinct antisocial symptoms. We found that both Deceitfulness and LPE symptoms were positively associated with Neurosynth meta-analytic term *Gain* (i.e., Ventral Striatum, frontopolar cortex, pgACC) (*r*s = 0.63–76) and negatively with faces (e.g., Amygdala, Fusiform gyrus, MCC). Furthermore, LPE symptoms were positively associated with *Reward* (i.e., Ventral Striatum, vmPFC, frontopolar cortex, and pgACC). Also, aggressivity and irresponsibility symptoms showed no significant association with Neurosynth terms.

### Examining the heterogeneity between lesion-based meta-analytic networks

Given that lesions are spatially distributed across ATL and mPFC regions, we sought to examine their heterogeneity in terms of co-activation mapping across fMRI tasks. As expected, analyses unveiled that lesion-based meta-analytic networks were spatially diverse and differed regarding their associated meta-analytic terms and receptor/transporter density maps. The different metrics used to assess the clustering solutions revealed that the four-cluster solution was the most optimal (see Supplementary material).

### Lesion-based co-activation group 1

**FIGURE 4:**
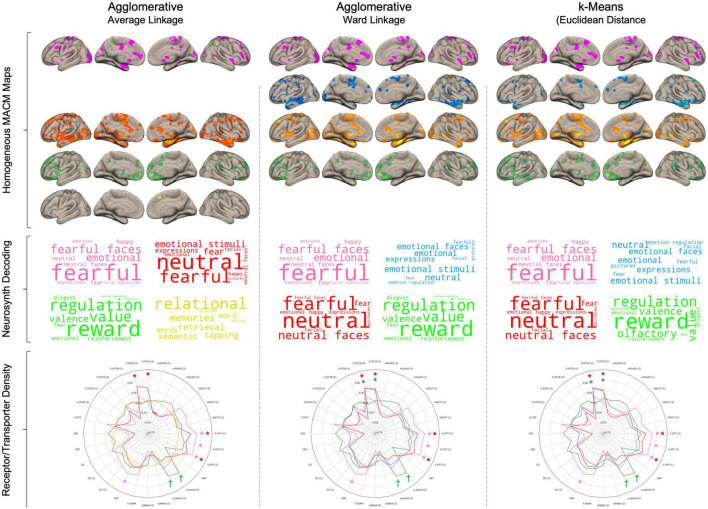
Results from the three different clustering algorithms. Rows represent cluster for each of the three algorithms. For each cluster, its respective brain network was produced by summing the thresholded and binarized lesion-specific networks that were assigned to the cluster. Wordclouds were generated by Neurosynth meta-analytic terms. Larger font represents stronger correlation. In Radar charts, Daggers (^†^) represent *p* < 0.001 uncorrected and asterisks (*) represent pFDR < 0.05.

This network only included lesions to the left amygdala (#15) and was reliably found by the three clustering algorithms (see Figure 4). It was principally characterized by deficient co-activity in the postcentral, thalamus, inferior frontal gyrus, right amygdala, caudate, and visual regions (Supplementary material). Functional decoding suggested that these maps were closely associated with emotion processing (i.e., fearful, emotional, happy, neutral) as well as face evaluation (i.e., fearful faces, neutral faces, expressions, facial expressions). Furthermore, this meta-analytic network was significantly associated with dopamine (*r* = 0.30) and serotonin (*rs* = 0.37–0.40) transporter maps.

### Lesion-based co-activation group 2

The second network was driven by temporal regions (#1 and #2) and was observed across the agglomerative clustering with euclidean distances (#1, #2, #3) and kMeans (#1, #2) but not the agglomerative clustering with spearman rank correlation. This network included the STG, insular cortex, fusiform face area, lateral PFC, and lateral OFC (see Supplementary material for complete results). Functional decoding suggested close associations with Neurosynth terms related to emotion processing in general (e.g., emotional stimuli, emotional faces, emotion regulation). This network was also only associated with 5-HT_1A_ receptor density maps (*rs* = 0.29–0.35).

### Lesion-based co-activation group 3

This network was mainly formed by lesion to the right amygdala (#12, #13) and was found by the three clustering algorithms. The agglomerative clustering using the spearman rank correlation found a broader network of lesions to temporal regions (#1, #2, #3, #12, #13) whereas the two others found only amygdala lesions (#12, #13). This network was mainly characterized by deficient co-activity in the bilateral amygdala, insular cortex, SMA, Fusiform face area, and midbrain, thalamus to a lesser extent (Supplementary material). This network showed prominent similarity with Neurosynth terms associated with emotional face processing (e.g., neutral, fearful, neutral faces, expressions). Furthermore, it was significantly associated with 5-HT_1A_ receptor density (*rs* = 29–0.37) as well as serotonin transporter (only the [^11^C]DASB radioligands *rs* = 0.25–0.27) maps.

### Lesion-based co-activation group 4

The fourth network included the remaining lesions which were mostly frontal (#6, #8, #9, #11, #14, #17). However, the included lesions in this group vary between the three method as the agglomerative clustering with spearman rank included six lesions (#6, #8, #9, #11, #14, #17), the one with ward linkage included 11 lesions (#4, #5, #6, #7, #8, #9, #10, #11, #14, #16, #17) and the kMeans included 12 lesions (#3, #4, #5, #6, #7, #8, #9, #10, #11, #14, #16, #17). Main aberrant co-activation was found in medial and lateral OFC, ventral to dorsal mPFC as well as posterior temporal gyrus. Neurosynth meta-analytic maps revealed an association with reward processing (e.g., reward, value, valence, reinforcement). This network was significantly associated with μ-opioid (*rs* = 0.36–0.43) and D_1_ ([^11^C]SCH23390 radioligand, *r* = 0.18) at an uncorrected threshold but did not survive FDR correction.

### Lesion-based co-activation group (other)

The agglomerative clustering with spearman rank distance revealed a cluster that was not observed by the other two cluster methods, which involved lesions to more dorsal PFC brain regions (#4, #5, #7, #10, #16). The associated network only included the pre-SMA. It showed similarity with various meta-analytic terms associated with memory and word processing and was not significantly associated with any receptor/transport density maps.

## Discussion

Recently, Darby et al. (20) used a lesion-to-voxel approach in healthy subjects at rest to examine the lesion brain network underpinning antisocial behaviors. They observed that this network principally overlapped with brain structures involved in moral decision-making. In complementarity, we sought to examine the neural architecture of lesions that were associated with antisocial behaviors during fMRI tasks. Indeed, we used images of brain lesions from 17 individuals (20) who were known to have committed antisocial behaviors after lesions (e.g., trauma, tumors) and conducted MACM using a database of 143 whole-brain fMRI experiments comprising more than 5,900 subjects exhibiting antisocial behaviors. Furthermore, we identified lesion networks that were specific to antisocial behaviors compared to 655 control lesions. We found a weak-to-moderate similarity in lesion-specific co-activation between antisocial and healthy subjects, indicating that compensation mechanisms may be linked with antisocial behaviors after lesions. The task-based lesion network associated with antisocial behaviors involved bilateral amygdala, medial and lateral OFC, supplementary motor area (SMA), ventro- and dorso-medial PFC, and fusiform area, which mainly correlated with emotion face processing and serotonin receptor (5-HT_1A_) and transporter (5-HTT) maps. However, we found only a weak overlap between the lesion MACM maps (∩ ≤ 29.4%), suggesting substantial heterogeneity between lesion networks. We further examined whether specific antisocial behaviors may explain this heterogeneity. First, we found evidence that specific antisocial symptoms were associated with distinct Neurosynth meta-analytic terms. Second, by using three different clustering algorithms, we observed that the 17 maps could be mainly separated into 4 homogenous groups: (1) lesion to the left amygdala which was associated with emotional face processing; (2) temporal lesions which were associated with general emotion processing, (3) right amygdala lesions which were associated with emotional face processing, and (4) frontal lesions which were correlated with reward processing. These groups exhibit different association patterns with serotoninergic, dopaminergic, and opioid systems.

As highlighted by Adolph and colleagues (21), individual differences are of utmost importance to the understanding of lesion brain networks underlying antisocial behaviors. Indeed, we found that the lesion-based MACM maps minimally overlapped at a group level (≤ 5 out of 17 lesions). This indicates a non-negligible level of heterogeneity between lesion-specific networks, but also questions the usefulness of studying lesion brain networks at a group level, as this approach is justified only by a broad phenotype that is *shared* between them (e.g., antisocial behavior). We thus performed additional analyses to better understand the interindividual variability concerning specific antisocial symptoms. We observed that antisocial symptoms were significantly associated with distinct Neurosynth meta-analytic terms. For instance, LPE and Deceitfulness were both positively associated with *Gain*. Deceitfulness and LPE are often co-occurring, both representing the interpersonal and affective facets underlying the Factor 1 of psychopathy (46). Thus, lesions that alter neural networks associated with *Gain* (e.g., ventral and dorsal striatum, frontopolar cortex, pgACC, middle frontal gyrus) may increase the risk for deceitfulness and LPE symptoms (e.g., conning others for personal profit, lying, lack of empathy, and remorse). Traits associated with factor 1 of psychopathy are often difficult to treat, but evidence nonetheless suggests that they might be more responsive to positive reinforcement strategies than punishment (47), potentially due to vulnerability toward gain and reward processes. Finally, although it was not statistically significant aggressivity/irritability symptoms showed opposite direction with meta-analytic terms gain (*r* = −0.18) and reward (*r* = −0.32) and positive association with faces (*r* = 0.21) and angry (*r* = 0.18). This opposite effect between antisocial behaviors is somewhat unsurprising given that aggressivity is more likely to be associated with negative emotional arousal than reward processes (48). These also concur with past evidence suggesting that aggressivity and other rule-breaking behaviors are distinct behavioral entities that are characterized by different etiological influences (49), developmental risk factors (50), and trajectories (51). Our results highlight the importance of studying specific antisocial behaviors in relationship with neurobiological markers.

In our study, we found that lesions that were linked to antisocial behaviors were mainly located in the mPFC and amygdala. Interestingly, past meta-analyses on healthy subjects found that both regions are frequently coactivating when performing various fMRI tasks such as morality (52), emotion processing (53), reward processing (54), and subjective value (55). However, we found that in antisocial subjects, lesions to these brain regions may alter distinct brain networks rather than a single common co-activation network. Indeed, by using a data-driven method, we observed four main homogeneous groups of lesion-based MACM maps which encompassed emotion face processing (Group 1 and Group 3: Amygdala, fusiform face area, thalamus, and visual regions), general emotion processing (Group 2: Superior Temporal Gyrus, Lateral PFC, Insula, MCC/SMA, Fusiform) and reward processing (Group 4: medial and lateral OFC, dorsal and ventral mPFC, and middle temporal gyrus). The four groups may largely depend on the location of brain lesions, namely the amygdala, temporal, and frontal. Indeed, these results concur with past lesion studies indicating that amygdala lesions may be selective to impairments in emotional face processing (56–61), whereas lesions to mOFC/vmPFC increase deficits in valuation and reward-guided decision-making (62–65). Interestingly, the four groups also differ in their spatial similarity with receptor maps. For instance, the task-based MACM maps of Group 1 (Left Amygdala) and Group 3 (Right Amygdala) were associated with serotonin transporter maps. This concurs with evidence supporting the role of 5-HTT in negative emotionality, neural reactivity of the amygdala (66) but also fearful face processing (67). We also found that Group 2 (Temporal) was associated with 5-HT_1A_ receptors, which support its role in aggressive and hostile behaviors (68–71), but also emotional lability in general (72). Finally, Group #4 (frontal lesions) rather showed a stronger association with μ-opioid receptors map. Indeed, μ-opioid receptors (73) are mainly located across the striatum (i.e., nucleus accumbens, globus pallidus, putamen, caudate) but also in vmPFC and medial OFC, supporting the role of reward processing in antisocial behaviors. Interestingly, the endogenous opioid system has recently been linked to several reward-related antisocial behaviors (e.g., manipulativeness), substance use, and sensation-seeking (74, 75), which supports our results regarding Deceitfulness and LPE symptoms. Overall, our results provide evidence of how different neurotransmitter systems may play prominent roles in the emergence of antisocial behaviors (14–16). Despite that the emphasis in literature is being mainly placed on dopaminergic and serotoninergic systems, the relationship between the opioid system and antisocial behaviors warrants further investigation.

Taking together, our results indicate that lesions to distinct brain regions may alter different brain networks underlying distinct neurocognitive processes which may increase the risk for distinct antisocial behaviors. Overall, we found that the brain lesions associated with antisocial behaviors were mainly located in the frontal and temporal lobes. Interestingly, these complex and heterogeneous interactions between brain regions and behaviors have also been reported in frontotemporal dementias, a clinical syndrome characterized by degeneration of prefrontal and/or temporal cortices. For instance, up to 57% of patients with frontotemporal dementias have reported antisocial behaviors [Average percentage across studies = 34%; (76)]. More precisely, patients with the frontal variant may exhibit a lack of empathy and emotional coldness, disinhibition, a significant reduction in agreeableness (i.e., callousness and distrustfulness), but also an increase in positive emotions (e.g., euphoric mood, exaggerated self-esteem) (77, 78). Moreover, some researchers have found that impairments in working memory are the most common deficit in the frontal variant (77). On the other hand, working memory tends to be preserved in the temporal lobe variant [Semantic variant: (78)], but patients may show impairments in object recognition, prosopagnosia, and deficits in emotional processing (77, 78). While brain-behavior relationships are complex, our findings tend to demonstrate that antisocial behaviors may emerge from four different brain mechanisms, largely depending on the location of the brain lesion. In the following years, future studies may seek to investigate the heterogeneity in neural processes associated with lesions as well as the interindividual variability in terms of clinical presentation.

## Limitations

There are a few limitations that need to be acknowledged. First, although we have conducted meta-analyses of aberrant co-activation using data from a large database of more than 5,900 antisocial subjects, only 17 lesion masks were used as seeds. Despite the small sample size involved, the clear advantage of this approach is that it allows the establishment of a clear temporal association between these 17 lesions and the subsequent emergence of antisocial behaviors. Second, we could not perform analyses specifically for each specific neurocognitive domain (i.e., positive valence, negative valence, social cognition, cognitive control), therefore we had to combine them and by doing so, it increased the heterogeneity in the co-activation network of a given seed. Nonetheless, this is a valid approach given that the standard MACM approach using the BrainMap database (25–27) is to meta-analyze all available experiments, regardless of the tasks. Also, our control lesions were stroke lesions. Although the sample size was large, we did not have any information on whether some patients developed aggressive behaviors or delinquency. As between 15 and 35% of poststroke patients may exhibit anger and agitation (79), comparing the antisocial lesions to it may have reduced the ability to find significant differences. Also, it would have been optimal to carefully match the 17 antisocial lesions with control lesions, antisocial subjects (without lesion), and healthy subjects (without lesion). Given that we used a meta-analytic method to investigate the lesioned network, future studies should seek to investigate this explicitly. Finally, we conducted clustering analyses using three different clustering methods to reduce biases due to the selection of clustering techniques. In meta-analytic neuroimaging literature, the most frequently used clustering based on the similarity of activated voxels is hierarchical clustering (44, 80, 81) and kMeans (54, 82). As there are numerous clustering methods and distance metrics, it is possible that our results may vary depending on the choice of other clustering methods (e.g., Affinity propagation, DBScan, etc.) or distance metrics (e.g., Manhattan, Minkowski, Jaccard, etc.).

## Conclusion

In sum, these results are of crucial importance given that it allows the understanding of individual variations in the emergence of antisocial behaviors after brain lesion. In other words, despite that all lesioned patients exhibited at least one antisocial behavior, they were all characterized by a different set of antisocial symptoms and a unique brain co-activation network. To reduce the heterogeneity between lesions, we first examined the clinical heterogeneity and found that Deceitfulness and LPE symptoms may be associated with neural maps related to Gain and Reward. Furthermore, we found homogeneous groups of lesion networks which may be associated with increased risk for antisocial behaviors. These were dependent on the lesion’s location and mainly encompassed emotional face processing, general emotion processing, and reward processing. Moreover, they show an association with distinct receptor/transport maps which may inform us on potential pharmacological treatments to reduce the risk for antisocial behaviors based on the lesion location. Given that the interaction between brain lesions and antisocial behaviors is complex (83), prospective studies are needed to support our results.

## Data availability statement

The raw data supporting the conclusions of this article will be made available by the authors, without undue reservation.

## Author contributions

JD and SP conceived and designed the study. JD conducted the analyses and wrote drafts of the manuscript. SP reviewed the drafts. Both authors approved the final version of the manuscript.

## Footnotes

1
https://github.com/JulDugre/AntisocialBrainDatabase

2
https://github.com/juryxy/JuSpace
