# Supplementary material for: The origins of evil: From lesions to the functional architecture of the antisocial brain
Source: Front Psychiatry. 2022 Oct 25;13:969206. doi: 10.3389/fpsyt.2022.969206 (PMC9640636; doi:10.3389/fpsyt.2022.969206)
Supplement: Supplementary file 1 [file Data_Sheet_1.PDF]

– SUPPLEMENTARY MATERIAL –

The origins of evil: from lesions to functional architecture of the antisocial brain.

Jules R. Dugré, MSc [1,2] & Stéphane Potvin, PhD [1,2]

[1] Research Center of the Institut Universitaire en Santé Mentale de Montréal, Montreal, Canada
[2] Department of Psychiatry and Addictology, Faculty of medicine, University of Montreal, Montreal, Canada

Corresponding authors

Jules Roger Dugré, PhD Candidate & Stéphane Potvin, PhD;
Research Center of the Institut Universitaire en Santé Mentale de Montréal; 7331 Hochelaga, Montreal, Quebec, Canada; H1N 3V2; Phone: 514-251-4015;
Email: jules.dugre@umontreal.ca / stephane.potvin@umontreal.ca

Table of Contents

Supplementary Table 1. Information about Neurotransmitter receptors and transporters included in the study....................................3

Supplementary Figure 1. Number of included neuroimaging studies per year (k=141) 4

Supplementary Table 2. Results from the Receptor/Transporter decoding of the thresholded MACM maps at a group-level................5

Supplementary Table 3. Results from the Neurosynth decoding of thresholded MACM maps at a group-level...................................6

Supplementary Table 4. Results from the associations between antisocial behaviors and Neurosynth Terms...................................7

Supplementary Figure 2. Metrics of the 2 to 5 cluster solutions for the three different cluster algorithms...8

Supplementary Table 5. Results from the lesion-based meta-analytic connectivity modelling for Group 1..9

Supplementary Table 6. Results from the lesion-based meta-analytic connectivity modelling for Group 2..10

Supplementary Table 7. Results from the lesion-based meta-analytic connectivity modelling for Group 3..11

Supplementary Table 8. Results from the lesion-based meta-analytic connectivity modelling for Group 4..12

Supplementary Table 9. Results from the Neurosynth decoding of Data-Driven Coactivation Groups for Agglomerative Clustering with Spearman Rank Distance...13

Supplementary Table 10. Results from the Neurosynth decoding of Data-Driven Coactivation Groups for Agglomerative Clustering with Euclidean Distance...14

Supplementary Table 11. Results from the Neurosynth decoding of Data-Driven Coactivation Groups for KMeans.........................15

Supplementary Table 12. Results from the Receptor/Transporter decoding of the Data-Driven Coactivation Groups averaged across Clustering Algorithms..16

List of the Included Studies..17

Supplementary Table 1. Information about Neurotransmitter receptors and transporters included in the study

Receptor/Transporter	Neurotransmitter	Tracer	N	References
VAChT (1)	acetylcholine	[^{18}F]FEOBV	6	Aghourian, 2017 [1]
VAChT (2)	acetylcholine	[^{18}F]FEOBV	4	see Hansen, 2021 [2]
VAChT (3)	acetylcholine	[^{18}F]FEOBV	5	Bédard, 2019 [3]
CB_1	cannabinoid	[^{11}C]OMAR	77	see Hansen, 2021 [2]
D_1	dopamine	[^{11}C]SCH23390	13	Kaller, 2017 [4]
D_2	dopamine	[^{11}C]raclopride	7	Alakurtti, 2015 [5]
D_2	dopamine	[11C]FLB-457	58	Jaworska, 2020 [6]
DAT	dopamine	[^{123}I]-FP-CIT	174	Dukart, 2018 [7]
Dopamine synthesis	dopamine	^{18}F-fluorodopa	12	García-Gómez, 2013 [8]
$GABA_A$ (1)	GABA	[^{11}C]flumazenil	6	Dukart, 2018 [7]
$GABA_A$ (2)	GABA	[^{11}C]flumazenil	16	Nørgaard, 2021 [9]
$mGluR_5$ (1)	glutamate	[^{11}C]ABP688	22	see Hansen, 2021 [2]
$mGluR_5$ (2)	glutamate	[^{11}C]ABP688	28	DuBois, 2016 [10]
$mGluR_5$ (3)	glutamate	[^{11}C]ABP688	73	Smart, 2019 [11]
NAT	noradrenaline	(*S*,*S*)-[11C]MRB	10	Hesse, 2017 [12]
μ-opioid (1)	opioid	[^{11}C]carfentanil	204	Kantonen, 2020 [13]
μ-opioid (2)	opioid	[^{11}C]carfentanil	39	Turtonen, 2021 [14]
$5\text{-}HT_{1A}$	serotonine	[^{11}C]WAY-100635	36	Savli, 2012 [15]
$5\text{-}HT_{1A}$	serotonine	[^{11}C]CUMI-101	8	Beliveau, 2017 [16]
$5\text{-}HT_{1B}$	serotonine	[^{11}C]P943	22	Savli, 2012 [15]
$5\text{-}HT_{1B}$	serotonine	[^{11}C]AZ10419369	36	Beliveau, 2017 [16]
$5\text{-}HT_{2A}$	serotonine	[^{18}F]ALT	19	Savli, 2012 [15]
$5\text{-}HT_{2a}$	serotonine	[^{11}C]Cimbi-36	29	Beliveau, 2017 [16]
$5\text{-}HT_4$	serotonine	[^{11}C]SB207145	59	Beliveau, 2017 [16]
5-HTT	serotonine	[^{11}C]MADAM	10	https://www.nitrc.org/projects/ki-5htt
5-HTT (1)	serotonine	[^{11}C]DASB	30	Savli, 2012 [15]
5-HTT (2)	serotonine	[^{11}C]DASB	100	Beliveau, 2017 [16]
CBF	-	*Arterial spin labeling	30	Holiga, 2018 [17]

Note.

1. Aghourian M, Legault-Denis C, Soucy JP, et al. Quantification of brain cholinergic denervation in Alzheimer's disease using PET imaging with [(18)F]-FEOBV. *Mol Psychiatry.* 2017;22(11):1531-1538.
2. Hansen JY, Shafiei G, Markello RD, et al. Mapping neurotransmitter systems to the structural and functional organization of the human neocortex. *Biorxiv.* 2021.
3. Bedard MA, Aghourian M, Legault-Denis C, et al. Brain cholinergic alterations in idiopathic REM sleep behaviour disorder: a PET imaging study with (18)F-FEOBV. *Sleep Med.* 2019;58:35-41.
4. Kaller S, Rullmann M, Patt M, et al. Test-retest measurements of dopamine D(1)-type receptors using simultaneous PET/MRI imaging. *Eur J Nucl Med Mol Imaging.* 2017;44(6):1025-1032.
5. Alakurtti K, Johansson JJ, Joutsa J, et al. Long-term test-retest reliability of striatal and extrastriatal dopamine D2/3 receptor binding: study with [(11)C]raclopride and high-resolution PET. *J Cereb Blood Flow Metab.* 2015;35(7):1199-1205.
6. Jaworska N, Cox SML, Tippler M, et al. Extra-striatal D(2/3) receptor availability in youth at risk for addiction. *Neuropsychopharmacology.* 2020;45(9):1498-1505.
7. Dukart J, Holiga Š, Chatham C, et al. Cerebral blood flow predicts differential neurotransmitter activity. *Scientific Reports.* 2018;8(1):4074.
8. García-Gómez FJ, García-Solís D, Luis-Simón FJ, et al. [Elaboration of the SPM template for the standardization of SPECT images with 123I-Ioflupane]. *Rev Esp Med Nucl Imagen Mol.* 2013;32(6):350-356.
9. Nørgaard M, Beliveau V, Ganz M, et al. A high-resolution in vivo atlas of the human brain's benzodiazepine binding site of GABA(A) receptors. *Neuroimage.* 2021;232:117878.
10. DuBois JM, Rousset OG, Rowley J, et al. Characterization of age/sex and the regional distribution of mGluR5 availability in the healthy human brain measured by high-resolution [(11)C]ABP688 PET. *Eur J Nucl Med Mol Imaging.* 2016;43(1):152-162.
11. Smart K, Cox SML, Scala SG, et al. Sex differences in [(11)C]ABP688 binding: a positron emission tomography study of mGlu5 receptors. *Eur J Nucl Med Mol Imaging.* 2019;46(5):1179-1183.
12. Hesse S, Becker GA, Rullmann M, et al. Central noradrenaline transporter availability in highly obese, non-depressed individuals. *Eur J Nucl Med Mol Imaging.* 2017;44(6):1056-1064.
13. Kantonen T, Karjalainen T, Isojärvi J, et al. Interindividual variability and lateralization of μ-opioid receptors in the human brain. *NeuroImage.* 2020;217:116922.
14. Turtonen O, Saarinen A, Nummenmaa L, et al. Adult Attachment System Links With Brain Mu Opioid Receptor Availability In Vivo. *Biol Psychiatry Cogn Neurosci Neuroimaging.* 2021;6(3):360-369.
15. Savli M, Bauer A, Mitterhauser M, et al. Normative database of the serotonergic system in healthy subjects using multi-tracer PET. *NeuroImage.* 2012;63(1):447-459.
16. Beliveau V, Ganz M, Feng L, et al. A High-Resolution In Vivo Atlas of the Human Brain's Serotonin System. *J Neurosci.* 2017;37(1):120-128.
17. Holiga Š, Sambataro F, Luzy C, et al. Test-retest reliability of task-based and resting-state blood oxygen level dependence and cerebral blood flow measures. *PLOS ONE.* 2018;13(11):e0206583.

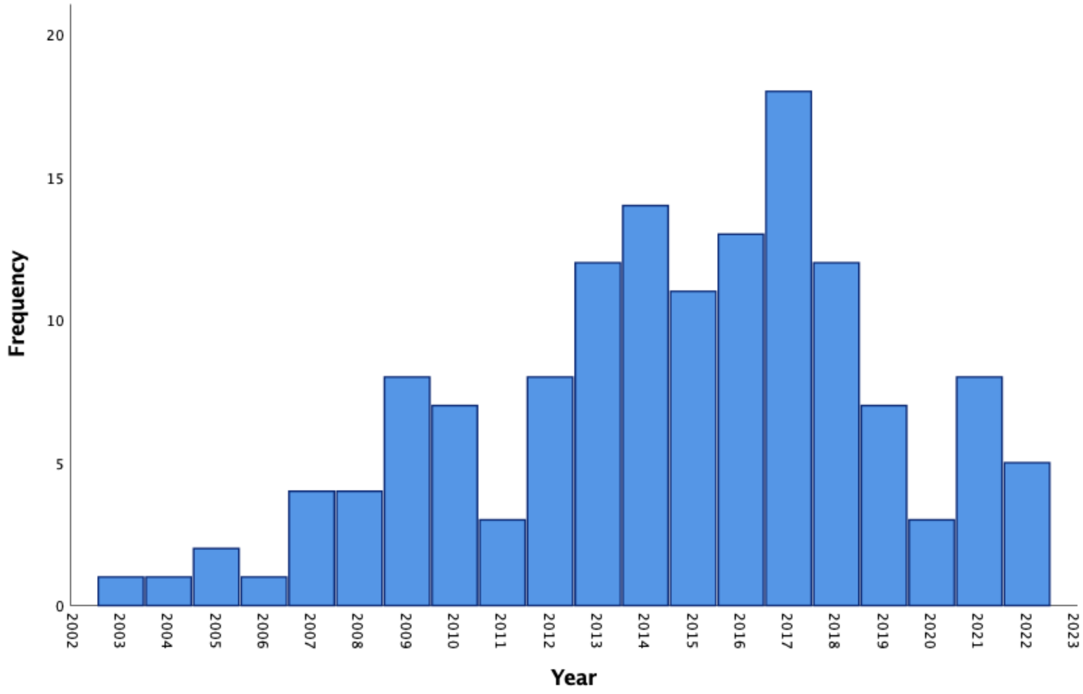

Supplementary Figure 1. Number of included neuroimaging studies per year (Antisocial Brain Database, k=141).

Supplementary Table 2. Results from the Receptor/Transporter decoding of the thresholded MACM maps at a group-level

Receptor/Transporter	Mean Fisher's z (Spearman r)	p-exact	p-FDR
$5\text{-}HT_{1A}$ $[^{11}C]$WAY-100635	0.2605	0.021	0.053
**$5\text{-}HT_{1A}$ $[^{11}C]$CUMI-101**	**0.3372**	**0.010**	**0.031**
$5\text{-}HT_{1B}$ $[^{11}C]$P943	-0.3264	0.009	0.031
$5\text{-}HT_{1B}$ $[^{11}C]$AZ10419369	-0.0069	0.940	0.940
$5\text{-}HT_{2A}$ $[^{18}F]$ALT	-0.323	0.002	0.017
$5\text{-}HT_{2a}$ $[^{11}C]$Cimbi-36	-0.2466	0.036	0.072
$5\text{-}HT_4$ $[^{11}C]$SB207145	0.1032	0.219	0.340
CB_1 $[^{11}C]$OMAR	-0.3827	0.008	0.031
CBF	-0.3868	0.003	0.017
D_1 $[^{11}C]$SCH23390	0.0184	0.816	0.887
D_2 $[^{11}C]$raclopride	-0.0346	0.702	0.819
D_2 [11C]FLB-457	0.0836	0.353	0.520
DAT $[^{123}I]$-FP-CIT	0.1984	0.031	0.067
^{18}F-DOPA	0.1274	0.150	0.247
$GABA_A$ $[^{11}C]$flumazenil (1)	-0.2966	0.002	0.017
$GABA_A$ $[^{11}C]$flumazenil (2)	-0.3333	0.016	0.045
μ-opioid $[^{11}C]$carfentanil (1)	0.1255	0.401	0.561
μ-opioid $[^{11}C]$carfentanil (2)	0.1062	0.507	0.677
NAT (S,S)-[11C]MRB	-0.0096	0.921	0.940
**5-HTT $[^{11}C]$DASB (1)**	**0.3813**	**0.001**	**0.017**
**5-HTT $[^{11}C]$MADAM**	**0.2708**	**0.010**	**0.031**
**5-HTT $[^{11}C]$DASB (2)**	**0.3239**	**0.003**	**0.017**
VAChT $[^{18}F]$FEOBV (1)	0.0194	0.823	0.887
VAChT $[^{18}F]$FEOBV (2)	0.0375	0.698	0.819
VAChT $[^{18}F]$FEOBV (3)	0.0554	0.535	0.681
$mGluR_5$ $[^{11}C]$ABP688 (1)	-0.2133	0.081	0.142
$mGluR_5$ $[^{11}C]$ABP688 (2)	-0.271	0.031	0.067
$mGluR_5$ $[^{11}C]$ABP688 (3)	-0.2419	0.063	0.117

Note.

Supplementary Table 3. Results from the Neurosynth decoding of thresholded MACM maps at a group-level

Neurosynth Terms	*r*
Neutral	0.308
Emotional	0.288
Fearful	0.288
Fear	0.284
Neutral Faces	0.264
Emotional Stimuli	0.261
Happy	0.260
Expressions	0.253
Pictures	0.251
Facial	0.247

Note.

Supplementary Table 4. Results from the associations between antisocial behaviors and similarity with Neurosynth Terms

Behaviors	Neurosynth Terms	Pearson Correlation	p-exact	p-FDR	Lesions	Pearson Correlation	p-exact	p-FDR
Aggressivity	Angry	0.184	0.478	0.956	Deceitfulness	-0.264	0.307	0.409
	Demands	0.026	0.920	1.000		0.079	0.763	0.763
	Fear	0.079	0.763	1.000		-0.29	0.259	0.409
	Gain	-0.184	0.478	0.956		.632	0.006	0.048
	Reward	-0.316	0.216	0.956		0.29	0.259	0.409
	Task	-0.132	0.614	0.982		0.158	0.544	0.622
	Faces	0.211	0.417	0.956		-.553	0.021	0.084
	Happy	0.000	1.000	1.000		-0.369	0.145	0.387
Irresponsibility	Angry	-0.217	0.404	0.462	LPE	-0.439	0.078	0.125
	Demands	0.265	0.305	0.407		-0.024	0.926	0.926
	Fear	-0.144	0.580	0.580		-0.390	0.121	0.161
	Gain	0.313	0.222	0.407		**0.756**	**0.001**	**0.008**
	Reward	0.289	0.261	0.407		0.488	0.047	0.094
	Task	0.361	0.155	0.407		0.220	0.397	0.454
	Faces	-0.481	0.051	0.407		-0.610	0.009	0.036
	Happy	-0.289	0.261	0.407		-0.488	0.047	0.094

Note.

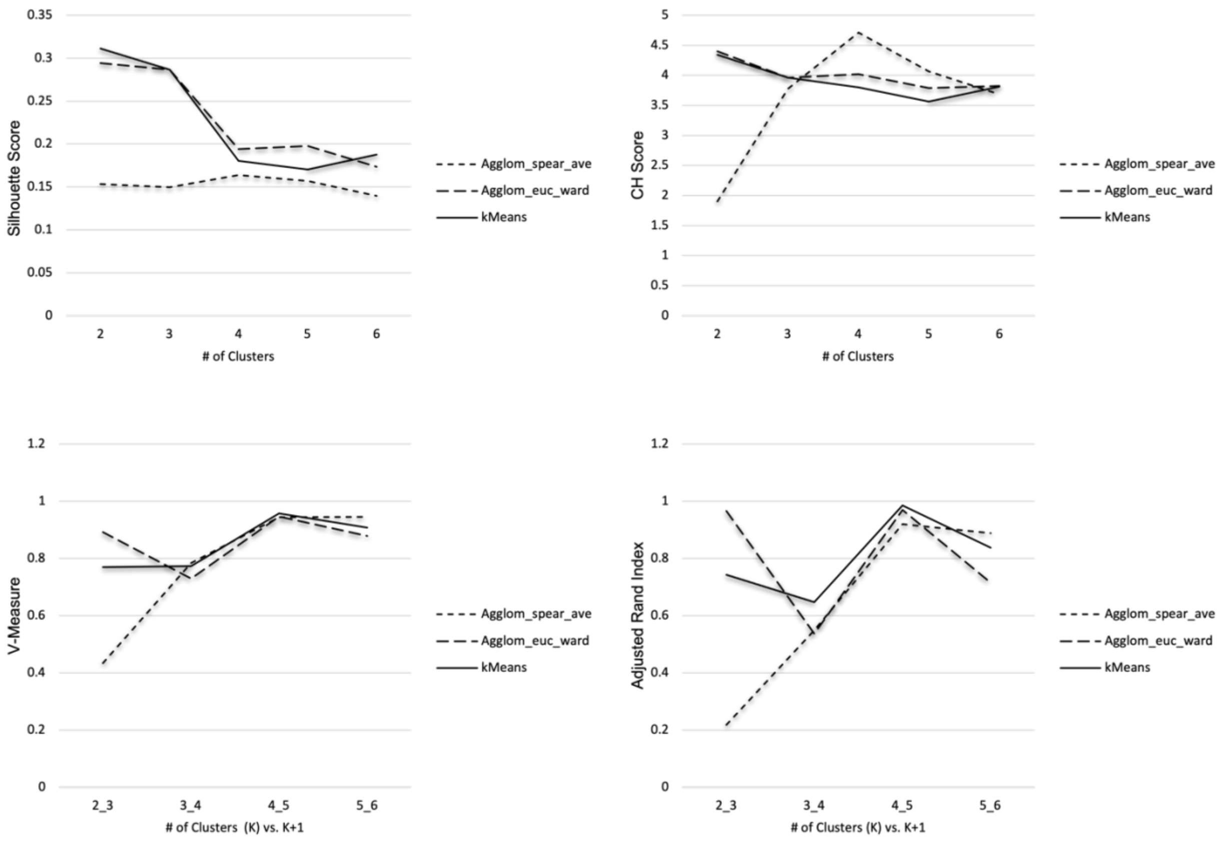

Supplementary Figure 2. Metrics of the 2 to 5 cluster solutions for the three different cluster algorithms.

Supplementary Table 5. Results from the lesion-based meta-analytic connectivity modelling for Group 1 (#15)

Regions	MNI coordinates			Z-score
	x	y	z	
HIP/AMY	-26	-6	-28	3.72
postcentral	-44	-10	26	3.35
thalamus	-16	-20	-4	3.24
IFG	40	34	6	3.24
hOc2 (V2)	6	-90	14	3.19
AMY	14	-8	-12	3.04
Caudate	-16	16	16	2.95
Caudate	10	4	4	2.81
hOc4lp	-32	-90	-6	2.75

Note. The overlap between task-based MACM images was performed by overlapping thresholded and binarized images. Only regions showing equal or more than 4 peaks are reported.

Supplementary Table 6. Results from the lesion-based meta-analytic connectivity modelling for Group 2

region	MNI Coordinates			Overlap within-cluster (%)		
	x	y	z	Agglom1	Agglom2	kMeans
STG	42	-4	-20	NA	100%	100%
Temporal Pole	44	16	-16	NA	100%	100%
Fusiform Face Area	-38	-42	-24	NA	66%	100%
Insular cortex	-38	-12	-10	NA	100%	100%
lateral OFC	-32	50	-8	NA	100%	100%
inferior LOC	-38	-80	4	NA	66%	-
lateral PFC	36	50	16	NA	66%	100%
Postcentral	-54	-22	36	NA	66%	-
lateral PFC	-34	40	36	NA	66%	100%
MCC	-4	2	38	NA	66%	-
SMA	-14	0	56	NA	66%	-

Note.

Supplementary Table 7. Results from the lesion-based meta-analytic connectivity modelling for Group 3

	MNI Coordinates			Overlap within-cluster (%)		
region	x	y	z	Agglom1	Agglom2	kMeans
AMY	-26	3	-26	80.0%	100%	100%
Insular Cortex	-38	-10	-10	80.0%	100%	100%
SMA	-14	0	56	80.0%	100%	100%
Fusiform Face Area	40	-18	-30	80.0%	100%	100%
inferior LOC (BA 19)	-38	-78	4	80.0%	100%	100%
AMY	24	-22	-20	60.0%	100%	100%
Midbrain	2	-12	-8	40.0%	100%	100%
Thalamus	-14	-20	-2	40.0%	100%	100%
dmPFC	-16	34	38	40.0%	100%	100%
Precentral	48	-2	38	40.0%	100%	100%

Note.

Supplementary Table 8. Results from the lesion-based meta-analytic connectivity modelling for Group 4

region	MNI Coordinates			Overlap within-cluster (%)		
	x	y	z	C1	C2	C3
mOFC	-18	24	-16	66.67%	36.36%	33.33%
vmPFC	14	54	-4	66.67%	36.36%	33.33%
lateral OFC	28	40	-16	50.00%	27.27%	25.00%
mPFC	-16	50	2	50.00%	27.27%	25.00%
dmPFC	-18	36	52	66.67%	36.36%	33.33%
dmPFC	-16	26	64	50.00%	27.27%	25.00%
posterior middle temporal cortex	-51	-73	23	33.33%	18.18%	16.67%
lateral OFC	-49	37	-15	33.33%	18.18%	16.67%
Temporal gyrus (BA 21-37)	-49	-42	-2	33.33%	18.18%	16.67%

Note.

Supplementary Table 9. Results from the Neurosynth decoding of Data-Driven Coactivation Groups for Agglomerative Clustering with Spearman Rank Distance

Coactivation Groups	Neurosynth Terms	r	Coactivation Groups	Neurosynth Terms	r
Group 1	fearful	0.190	Group 2		
	fearful faces	0.179			
	emotional	0.170			
	neutral faces	0.168			
	happy	0.167			
	neutral	0.164			
	fear	0.155			
	expressions	0.147			
	emotions	0.147			
	facial expressions	0.145			
Group 3	neutral	0.276	Group 4	reward	0.126
	fearful	0.260		regulation	0.107
	emotional stimuli	0.244		value	0.106
	fear	0.241		valence	0.104
	expressions	0.239		reinforcement	0.099
	emotional	0.238		disgust	0.098
	neutral faces	0.238		emotional	0.095
	facial	0.232		fear	0.091
	happy	0.231		emotion regulation	0.09
	pictures	0.228		affective	0.088

Note.

Supplementary Table 10. Results from the Neurosynth decoding of Data-Driven Coactivation Groups for Agglomerative Clustering with Euclidean Distance

Coactivation Groups	Neurosynth Terms	r	Coactivation Groups	Neurosynth Terms	r
Group 1	fearful	0.190	Group 2	emotional stimuli	0.148
	fearful faces	0.179		neutral	0.138
	emotional	0.170		emotional faces	0.118
	neutral faces	0.168		emotional	0.117
	happy	0.167		expressions	0.116
	neutral	0.164		emotion regulation	0.113
	fear	0.155		fearful	0.112
	expressions	0.147		facial	0.110
	emotions	0.147		pictures	0.110
	facial expressions	0.145		fear	0.107
Group 3	neutral	0.263	Group 4	reward	0.126
	fearful	0.260		regulation	0.107
	neutral faces	0.243		value	0.106
	fear	0.239		valence	0.104
	happy	0.233		reinforcement	0.099
	expressions	0.232		disgust	0.098
	emotional	0.229		emotional	0.095
	facial	0.226		fear	0.091
	fearful faces	0.224		emotion regulation	0.09
	pictures	0.221		affective	0.088

Note.

Supplementary Table 11. Results from the Neurosynth decoding of Data-Driven Coactivation Groups for KMeans

Coactivation Groups	Neurosynth Terms	r	Coactivation Groups	Neurosynth Terms	r
Group 1	fearful	0.190	Group 2	emotional stimuli	0.196
	fearful faces	0.179		neutral	0.191
	emotional	0.170		emotional faces	0.159
	neutral faces	0.168		expressions	0.156
	happy	0.167		emotional	0.154
	neutral	0.164		emotion regulation	0.152
	fear	0.155		fearful	0.151
	expressions	0.147		pictures	0.149
	emotions	0.147		facial	0.148
	facial expressions	0.145		fear	0.143
Group 3	neutral	0.263	Group 4	reward	0.098
	fearful	0.260		regulation	0.088
	neutral faces	0.243		olfactory	0.086
	fear	0.239		value	0.084
	happy	0.233		valence	0.082
	expressions	0.232		reinforcement	0.080
	emotional	0.229		emotional	0.078
	facial	0.226		disgust	0.078
	fearful faces	0.224		affective	0.073
	pictures	0.221		fear	0.073

Note.

Supplementary Table 12. Results from the Receptor/Transporter decoding of the Data-Driven Coactivation Groups averaged across Clustering Algorithms

Coactivation Groups	Receptor/Transporter	Mean Fisher's z (Spearman r)	p-exact	p-FDR	Coactivation Groups	Mean Fisher's z (Spearman r)	p-exact	p-FDR
Group 1	5-HT$_{1A}$ [^{11}C]WAY-100635	-0.008	0.941	0.941	Group 2	**0.285**	**0.003**	**0.042**
	5-HT$_{1A}$ [^{11}C]CUMI-101	0.135	0.202	0.252		**0.345**	**0.002**	**0.042**
	5-HT$_{1B}$ [^{11}C]P943	-0.201	0.061	0.095		-0.222	0.038	0.354
	5-HT$_{1B}$ [^{11}C]AZ10419369	0.077	0.347	0.388		0.047	0.602	0.757
	5-HT$_{2A}$ [^{18}F]ALT	-0.398	0.001	0.009		-0.060	0.531	0.757
	5-HT$_{2a}$ [^{11}C]Cimbi-36	-0.299	0.004	0.016		0.033	0.758	0.786
	5-HT$_4$ [^{11}C]SB207145	0.237	0.025	0.064		0.039	0.629	0.757
	CB$_1$ [^{11}C]OMAR	-0.289	0.009	0.031		-0.181	0.098	0.440
	CBF	-0.310	0.004	0.016		-0.161	0.110	0.440
	D$_1$ [^{11}C]SCH23390	0.103	0.193	0.252		-0.065	0.431	0.753
	D$_2$ [^{11}C]raclopride	0.145	0.096	0.134		-0.113	0.181	0.633
	D$_2$ [11C]FLB-457	0.202	0.049	0.082		-0.031	0.697	0.757
	DAT [^{123}I]-FP-CIT	**0.304**	**0.012**	**0.037**		0.011	0.899	0.899
	^{18}F-DOPA	0.231	0.035	0.075		0.032	0.703	0.757
	GABA$_A$ [^{11}C]flumazenil (1)	-0.353	0.001	0.009		-0.101	0.277	0.693
	GABA$_A$ [^{11}C]flumazenil (2)	-0.119	0.280	0.326		-0.134	0.218	0.660
	μ-opioid [^{11}C]carfentanil (1)	0.146	0.207	0.252		0.072	0.542	0.757
	μ-opioid [^{11}C]carfentanil (2)	0.084	0.462	0.497		0.057	0.687	0.757
	NAT *(S,S)*-[^{11}C]MRB	-0.005	0.940	0.941		0.059	0.512	0.757
	5-HTT [^{11}C]DASB (1)	**0.403**	**0.002**	**0.011**		0.148	0.104	0.440
	5-HTT [^{11}C]MADAM	**0.367**	**0.002**	**0.011**		0.035	0.680	0.757
	5-HTT [^{11}C]DASB (2)	**0.399**	**0.001**	**0.009**		0.154	0.087	0.440
	VAChT [^{18}F]FEOBV (1)	0.178	0.070	0.103		-0.095	0.236	0.660
	VAChT [^{18}F]FEOBV (2)	0.226	0.031	0.072		-0.081	0.341	0.693
	VAChT [^{18}F]FEOBV (3)	0.210	0.046	0.082		-0.068	0.424	0.753
	mGluR$_5$ [^{11}C]ABP688 (1)	-0.217	0.050	0.082		-0.101	0.321	0.693
	mGluR$_5$ [^{11}C]ABP688 (2)	-0.254	0.021	0.059		-0.092	0.347	0.693
	mGluR$_5$ [^{11}C]ABP688 (3)	-0.220	0.047	0.082		-0.073	0.488	0.757
Group 3	5-HT$_{1A}$ [^{11}C]WAY-100635	**0.292**	**0.005**	**0.034**	Group 4	0.037	0.725	0.881
	5-HT$_{1A}$ [^{11}C]CUMI-101	**0.365**	**0.008**	**0.034**		-0.034	0.764	0.881
	5-HT$_{1B}$ [^{11}C]P943	-0.371	0.006	0.034		0.079	0.526	0.881
	5-HT$_{1B}$ [^{11}C]AZ10419369	-0.089	0.306	0.480		0.091	0.320	0.746
	5-HT$_{2A}$ [^{18}F]ALT	-0.268	0.005	0.034		-0.038	0.676	0.881
	5-HT$_{2a}$ [^{11}C]Cimbi-36	-0.249	0.029	0.074		0.024	0.830	0.881
	5-HT$_4$ [^{11}C]SB207145	0.031	0.736	0.825		0.035	0.719	0.881
	CB$_1$ [^{11}C]OMAR	-0.482	0.001	0.028		0.184	0.167	0.578
	CBF	-0.397	0.002	0.028		0.024	0.849	0.881
	D$_1$ [^{11}C]SCH23390	-0.082	0.357	0.499		*0.183*	*0.045*	*0.252*
	D$_2$ [^{11}C]raclopride	-0.083	0.309	0.480		0.006	0.944	0.944
	D$_2$ [11C]FLB-457	0.001	0.998	0.998		0.102	0.263	0.669
	DAT [^{123}I]-FP-CIT	0.093	0.293	0.480		0.147	0.103	0.412
	^{18}F-DOPA	0.051	0.568	0.692		0.074	0.445	0.881
	GABA$_A$ [^{11}C]flumazenil (1)	-0.248	0.010	0.034		-0.022	0.805	0.881
	GABA$_A$ [^{11}C]flumazenil (2)	-0.235	0.070	0.131		-0.332	0.008	0.196
	μ-opioid [^{11}C]carfentanil (1)	-0.051	0.686	0.801		*0.357*	*0.028*	*0.196*
	μ-opioid [^{11}C]carfentanil (2)	-0.080	0.555	0.692		*0.431*	*0.026*	*0.196*
	NAT *(S,S)*-[^{11}C]MRB	0.081	0.355	0.499		-0.235	0.014	0.196
	5-HTT [^{11}C]DASB (1)	**0.269**	**0.011**	**0.034**		0.154	0.103	0.412
	5-HTT [^{11}C]MADAM	0.174	0.058	0.116		0.123	0.186	0.578
	5-HTT [^{11}C]DASB (2)	**0.250**	**0.009**	**0.034**		0.035	0.711	0.881
	VAChT [^{18}F]FEOBV (1)	-0.050	0.544	0.692		0.064	0.500	0.881
	VAChT [^{18}F]FEOBV (2)	-0.026	0.783	0.843		0.024	0.799	0.881
	VAChT [^{18}F]FEOBV (3)	-0.006	0.947	0.982		0.042	0.657	0.881
	mGluR$_5$ [^{11}C]ABP688 (1)	-0.258	0.033	0.077		0.136	0.251	0.669
	mGluR$_5$ [^{11}C]ABP688 (2)	-0.309	0.013	0.036		0.097	0.415	0.881
	mGluR$_5$ [^{11}C]ABP688 (3)	-0.256	0.038	0.082		0.038	0.750	0.881

Note.

List of Included Studies

1. Abe N, Greene JD, Kiehl KA. Reduced engagement of the anterior cingulate cortex in the dishonest decision-making of incarcerated psychopaths. *Soc Cogn Affect Neurosci.* 2018;13(8):797-807.
2. Aghajani M, Klapwijk ET, Andershed H, et al. Neural processing of socioemotional content in conduct-disordered juvenile offenders with limited prosocial emotions. *Prog Neuropsychopharmacol Biol Psychiatry.* 2021;105:110045.
3. Alia-Klein N, Wang GJ, Preston-Campbell RN, et al. Reactions to media violence: it's in the brain of the beholder. *PLoS One.* 2014;9(9):e107260.
4. Anderson NE, Maurer JM, Steele VR, Kiehl KA. Psychopathic traits associated with abnormal hemodynamic activity in salience and default mode networks during auditory oddball task. *Cogn Affect Behav Neurosci.* 2018;18(3):564-580.
5. Anderson NE, Steele VR, Maurer JM, et al. Differentiating emotional processing and attention in psychopathy with functional neuroimaging. *Cogn Affect Behav Neurosci.* 2017;17(3):491-515.
6. Banich MT, Crowley TJ, Thompson LL, et al. Brain activation during the Stroop task in adolescents with severe substance and conduct problems: A pilot study. *Drug Alcohol Depend.* 2007;90(2-3):175-182.
7. Birbaumer N, Veit R, Lotze M, et al. Deficient fear conditioning in psychopathy: a functional magnetic resonance imaging study. *Arch Gen Psychiatry.* 2005;62(7):799-805.
8. Bjork JM, Chen G, Smith AR, Hommer DW. Incentive-elicited mesolimbic activation and externalizing symptomatology in adolescents. *J Child Psychol Psychiatry.* 2010;51(7):827-837.
9. Bobes MA, Ostrosky F, Diaz K, et al. Linkage of functional and structural anomalies in the left amygdala of reactive-aggressive men. *Soc Cogn Affect Neurosci.* 2013;8(8):928-936.
10. Buades-Rotger M, Engelke C, Krämer UM. Trait and state patterns of basolateral amygdala connectivity at rest are related to endogenous testosterone and aggression in healthy young women. *Brain Imaging Behav.* 2019;13(2):564-576.
11. Bubenzer-Busch S, Herpertz-Dahlmann B, Kuzmanovic B, et al. Neural correlates of reactive aggression in children with attention-deficit/hyperactivity disorder and comorbid disruptive behaviour disorders. *Acta Psychiatr Scand.* 2016;133(4):310-323.
12. Caldwell BM, Harenski CL, Harenski KA, et al. Abnormal frontostriatal activity in recently abstinent cocaine users during implicit moral processing. *Front Hum Neurosci.* 2015;9:565.
13. Cardinale EM, Breeden AL, Robertson EL, Lozier LM, Vanmeter JW, Marsh AA. Externalizing behavior severity in youths with callous-unemotional traits corresponds to patterns of amygdala activity and connectivity during judgments of causing fear. *Dev Psychopathol.* 2018;30(1):191-201.
14. Cazala F, Harenski KA, Thornton DM, Kiehl KA, Harenski CL. Neural Correlates of Moral Judgment in Criminal Offenders with Sadistic Traits. *Arch Sex Behav.* 2021;50(5):2163-2171.

15. Coccaro EF, McCloskey MS, Fitzgerald DA, Phan KL. Amygdala and orbitofrontal reactivity to social threat in individuals with impulsive aggression. *Biol Psychiatry.* 2007;62(2):168-178.
16. Cohn MD, Popma A, van den Brink W, et al. Fear conditioning, persistence of disruptive behavior and psychopathic traits: an fMRI study. *Transl Psychiatry.* 2013;3(10):e319.
17. Cohn MD, van Lith K, Kindt M, et al. Fear extinction, persistent disruptive behavior and psychopathic traits: fMRI in late adolescence. *Soc Cogn Affect Neurosci.* 2016;11(7):1027-1035.
18. Contreras-Rodríguez O, Pujol J, Batalla I, et al. Disrupted neural processing of emotional faces in psychopathy. *Soc Cogn Affect Neurosci.* 2014;9(4):505-512.
19. Contreras-Rodríguez O, Pujol J, Batalla I, et al. Functional Connectivity Bias in the Prefrontal Cortex of Psychopaths. *Biol Psychiatry.* 2015;78(9):647-655.
20. Cope LM, Vincent GM, Jobelius JL, Nyalakanti PK, Calhoun VD, Kiehl KA. Psychopathic traits modulate brain responses to drug cues in incarcerated offenders. *Front Hum Neurosci.* 2014;8:87.
21. Crowley TJ, Dalwani MS, Mikulich-Gilbertson SK, et al. Risky decisions and their consequences: neural processing by boys with Antisocial Substance Disorder. *PLoS One.* 2010;5(9):e12835.
22. da Cunha-Bang S, Fisher PM, Hjordt LV, et al. Violent offenders respond to provocations with high amygdala and striatal reactivity. *Soc Cogn Affect Neurosci.* 2017;12(5):802-810.
23. Decety J, Chen C, Harenski CL, Kiehl KA. Socioemotional processing of morally-laden behavior and their consequences on others in forensic psychopaths. *Hum Brain Mapp.* 2015;36(6):2015-2026.
24. Decety J, Michalska KJ, Akitsuki Y, Lahey BB. Atypical empathic responses in adolescents with aggressive conduct disorder: a functional MRI investigation. *Biol Psychol.* 2009;80(2):203-211.
25. Decety J, Skelly L, Yoder KJ, Kiehl KA. Neural processing of dynamic emotional facial expressions in psychopaths. *Soc Neurosci.* 2014;9(1):36-49.
26. Decety J, Skelly LR, Kiehl KA. Brain response to empathy-eliciting scenarios involving pain in incarcerated individuals with psychopathy. *JAMA Psychiatry.* 2013;70(6):638-645.
27. Deeley Q, Daly E, Surguladze S, et al. Facial emotion processing in criminal psychopathy. Preliminary functional magnetic resonance imaging study. *Br J Psychiatry.* 2006;189:533-539.
28. Deming P, Dargis M, Haas BW, et al. Psychopathy is associated with fear-specific reductions in neural activity during affective perspective-taking. *Neuroimage.* 2020;223:117342.
29. Deming P, Philippi CL, Wolf RC, Dargis M, Kiehl KA, Koenigs M. Psychopathic traits linked to alterations in neural activity during personality judgments of self and others. *Neuroimage Clin.* 2018;18:575-581.
30. Dong D, Ming Q, Wang X, et al. Temporoparietal Junction Hypoactivity during Pain-Related Empathy Processing in Adolescents with Conduct Disorder. *Front Psychol.* 2016;7:2085.

31. Ewbank MP, Passamonti L, Hagan CC, Goodyer IM, Calder AJ, Fairchild G. Psychopathic traits influence amygdala-anterior cingulate cortex connectivity during facial emotion processing. *Soc Cogn Affect Neurosci.* 2018;13(5):525-534.
32. Fairchild G, Hagan CC, Passamonti L, Walsh ND, Goodyer IM, Calder AJ. Atypical neural responses during face processing in female adolescents with conduct disorder. *J Am Acad Child Adolesc Psychiatry.* 2014;53(6):677-687.e675.
33. Fede SJ, Borg JS, Nyalakanti PK, et al. Distinct neuronal patterns of positive and negative moral processing in psychopathy. *Cogn Affect Behav Neurosci.* 2016;16(6):1074-1085.
34. Fehlbaum LV, Raschle NM, Menks WM, et al. Altered Neuronal Responses During an Affective Stroop Task in Adolescents With Conduct Disorder. *Front Psychol.* 2018;9:1961.
35. Finger EC, Marsh A, Blair KS, et al. Impaired functional but preserved structural connectivity in limbic white matter tracts in youth with conduct disorder or oppositional defiant disorder plus psychopathic traits. *Psychiatry Res.* 2012;202(3):239-244.
36. Finger EC, Marsh AA, Blair KS, et al. Disrupted reinforcement signaling in the orbitofrontal cortex and caudate in youths with conduct disorder or oppositional defiant disorder and a high level of psychopathic traits. *Am J Psychiatry.* 2011;168(2):152-162.
37. Fullam RS, McKie S, Dolan MC. Psychopathic traits and deception: functional magnetic resonance imaging study. *Br J Psychiatry.* 2009;194(3):229-235.
38. Gan G, Preston-Campbell RN, Moeller SJ, et al. Reward vs. Retaliation-the Role of the Mesocorticolimbic Salience Network in Human Reactive Aggression. *Front Behav Neurosci.* 2016;10:179.
39. Gatzke-Kopp LM, Beauchaine TP, Shannon KE, et al. Neurological correlates of reward responding in adolescents with and without externalizing behavior disorders. *J Abnorm Psychol.* 2009;118(1):203-213.
40. Geurts DE, von Borries K, Volman I, Bulten BH, Cools R, Verkes RJ. Neural connectivity during reward expectation dissociates psychopathic criminals from non-criminal individuals with high impulsive/antisocial psychopathic traits. *Soc Cogn Affect Neurosci.* 2016;11(8):1326-1334.
41. Glenn AL, Han H, Yang Y, Raine A, Schug RA. Associations between psychopathic traits and brain activity during instructed false responding. *Psychiatry Res Neuroimaging.* 2017;266:123-137.
42. Gregory S, Blair RJ, Ffytche D, et al. Punishment and psychopathy: a case-control functional MRI investigation of reinforcement learning in violent antisocial personality disordered men. *Lancet Psychiatry.* 2015;2(2):153-160.
43. Han T, Alders GL, Greening SG, Neufeld RW, Mitchell DG. Do fearful eyes activate empathy-related brain regions in individuals with callous traits? *Soc Cogn Affect Neurosci.* 2012;7(8):958-968.
44. Harenski CL, Edwards BG, Harenski KA, Kiehl KA. Neural correlates of moral and non-moral emotion in female psychopathy. *Front Hum Neurosci.* 2014;8:741.
45. Harenski CL, Harenski KA, Kiehl KA. Neural processing of moral violations among incarcerated adolescents with psychopathic traits. *Dev Cogn Neurosci.* 2014;10:181-189.
46. Harenski CL, Harenski KA, Shane MS, Kiehl KA. Aberrant neural processing of moral violations in criminal psychopaths. *J Abnorm Psychol.* 2010;119(4):863-874.

47. Harenski CL, Thornton DM, Harenski KA, Decety J, Kiehl KA. Increased frontotemporal activation during pain observation in sexual sadism: preliminary findings. *Arch Gen Psychiatry.* 2012;69(3):283-292.
48. Heesink L, Edward Gladwin T, Terburg D, van Honk J, Kleber R, Geuze E. Proximity alert! Distance related cuneus activation in military veterans with anger and aggression problems. *Psychiatry Res Neuroimaging.* 2017;266:114-122.
49. Herpertz SC, Huebner T, Marx I, et al. Emotional processing in male adolescents with childhood-onset conduct disorder. *J Child Psychol Psychiatry.* 2008;49(7):781-791.
50. Holz NE, Boecker-Schlier R, Buchmann AF, et al. Ventral striatum and amygdala activity as convergence sites for early adversity and conduct disorder. *Soc Cogn Affect Neurosci.* 2017;12(2):261-272.
51. Hosking JG, Kastman EK, Dorfman HM, et al. Disrupted Prefrontal Regulation of Striatal Subjective Value Signals in Psychopathy. *Neuron.* 2017;95(1):221-231.e224.
52. Hwang S, Nolan ZT, White SF, Williams WC, Sinclair S, Blair RJ. Dual neurocircuitry dysfunctions in disruptive behavior disorders: emotional responding and response inhibition. *Psychol Med.* 2016;46(7):1485-1496.
53. Jones AP, Laurens KR, Herba CM, Barker GJ, Viding E. Amygdala hypoactivity to fearful faces in boys with conduct problems and callous-unemotional traits. *Am J Psychiatry.* 2009;166(1):95-102.
54. Kalnin AJ, Edwards CR, Wang Y, et al. The interacting role of media violence exposure and aggressive-disruptive behavior in adolescent brain activation during an emotional Stroop task. *Psychiatry Res.* 2011;192(1):12-19.
55. Kärgel C, Massau C, Weiß S, et al. Evidence for superior neurobiological and behavioral inhibitory control abilities in non-offending as compared to offending pedophiles. *Hum Brain Mapp.* 2017;38(2):1092-1104.
56. Kärgel C, Massau C, Weiß S, Walter M, Kruger TH, Schiffer B. Diminished functional connectivity on the road to child sexual abuse in pedophilia. *J Sex Med.* 2015;12(3):783-795.
57. Kiehl KA, Smith AM, Mendrek A, Forster BB, Hare RD, Liddle PF. Temporal lobe abnormalities in semantic processing by criminal psychopaths as revealed by functional magnetic resonance imaging. *Psychiatry Res.* 2004;130(1):27-42.
58. Klapwijk ET, Aghajani M, Colins OF, et al. Different brain responses during empathy in autism spectrum disorders versus conduct disorder and callous-unemotional traits. *J Child Psychol Psychiatry.* 2016;57(6):737-747.
59. Klapwijk ET, Lelieveld GJ, Aghajani M, et al. Fairness decisions in response to emotions: a functional MRI study among criminal justice-involved boys with conduct disorder. *Soc Cogn Affect Neurosci.* 2016;11(4):674-682.
60. Kneer J, Borchardt V, Kärgel C, et al. Diminished fronto-limbic functional connectivity in child sexual offenders. *J Psychiatr Res.* 2019;108:48-56.
61. Konzok J, Henze GI, Peter H, et al. Externalizing behavior in healthy young adults is associated with lower cortisol responses to acute stress and altered neural activation in the dorsal striatum. *Psychophysiology.* 2021;58(12):e13936.

62. Korponay C, Pujara M, Deming P, et al. Impulsive-antisocial psychopathic traits linked to increased volume and functional connectivity within prefrontal cortex. *Soc Cogn Affect Neurosci.* 2017;12(7):1169-1178.
63. Korponay C, Pujara M, Deming P, et al. Impulsive-antisocial dimension of psychopathy linked to enlargement and abnormal functional connectivity of the striatum. *Biol Psychiatry Cogn Neurosci Neuroimaging.* 2017;2(2):149-157.
64. Kose S, Steinberg JL, Moeller FG, et al. Neural correlates of impulsive aggressive behavior in subjects with a history of alcohol dependence. *Behav Neurosci.* 2015;129(2):183-196.
65. Kumari V, Das M, Taylor PJ, et al. Neural and behavioural responses to threat in men with a history of serious violence and schizophrenia or antisocial personality disorder. *Schizophr Res.* 2009;110(1-3):47-58.
66. Larson CL, Baskin-Sommers AR, Stout DM, et al. The interplay of attention and emotion: top-down attention modulates amygdala activation in psychopathy. *Cogn Affect Behav Neurosci.* 2013;13(4):757-770.
67. Lockwood PL, Sebastian CL, McCrory EJ, et al. Association of callous traits with reduced neural response to others' pain in children with conduct problems. *Curr Biol.* 2013;23(10):901-905.
68. Lozier LM, Cardinale EM, VanMeter JW, Marsh AA. Mediation of the relationship between callous-unemotional traits and proactive aggression by amygdala response to fear among children with conduct problems. *JAMA Psychiatry.* 2014;71(6):627-636.
69. Marsh AA, Finger EC, Fowler KA, et al. Empathic responsiveness in amygdala and anterior cingulate cortex in youths with psychopathic traits. *J Child Psychol Psychiatry.* 2013;54(8):900-910.
70. Marsh AA, Finger EC, Fowler KA, et al. Reduced amygdala-orbitofrontal connectivity during moral judgments in youths with disruptive behavior disorders and psychopathic traits. *Psychiatry Res.* 2011;194(3):279-286.
71. Marsh AA, Finger EC, Mitchell DG, et al. Reduced amygdala response to fearful expressions in children and adolescents with callous-unemotional traits and disruptive behavior disorders. *Am J Psychiatry.* 2008;165(6):712-720.
72. Massau C, Kärgel C, Weiß S, et al. Neural correlates of moral judgment in pedophilia. *Soc Cogn Affect Neurosci.* 2017;12(9):1490-1499.
73. Meffert H, Gazzola V, den Boer JA, Bartels AA, Keysers C. Reduced spontaneous but relatively normal deliberate vicarious representations in psychopathy. *Brain.* 2013;136(Pt 8):2550-2562.
74. Michalska KJ, Zeffiro TA, Decety J. Brain response to viewing others being harmed in children with conduct disorder symptoms. *J Child Psychol Psychiatry.* 2016;57(4):510-519.
75. Mier D, Haddad L, Diers K, Dressing H, Meyer-Lindenberg A, Kirsch P. Reduced embodied simulation in psychopathy. *World J Biol Psychiatry.* 2014;15(6):479-487.
76. Müller JL, Sommer M, Döhnel K, Weber T, Schmidt-Wilcke T, Hajak G. Disturbed prefrontal and temporal brain function during emotion and cognition interaction in criminal psychopathy. *Behav Sci Law.* 2008;26(1):131-150.
77. Müller JL, Sommer M, Wagner V, et al. Abnormalities in emotion processing within cortical and subcortical regions in criminal psychopaths: evidence from a functional

magnetic resonance imaging study using pictures with emotional content. *Biol Psychiatry.* 2003;54(2):152-162.
78. Murray L, Lopez-Duran NL, Mitchell C, Monk CS, Hyde LW. Antisocial behavior is associated with reduced frontoparietal activity to loss in a population-based sample of adolescents. *Psychol Med.* 2022:1-9.
79. O'Nions E, Sebastian CL, McCrory E, Chantiluke K, Happé F, Viding E. Neural bases of Theory of Mind in children with autism spectrum disorders and children with conduct problems and callous-unemotional traits. *Dev Sci.* 2014;17(5):786-796.
80. Osumi T, Nakao T, Kasuya Y, Shinoda J, Yamada J, Ohira H. Amygdala dysfunction attenuates frustration-induced aggression in psychopathic individuals in a non-criminal population. *J Affect Disord.* 2012;142(1-3):331-338.
81. Pape L, van Lith K, Veltman D, et al. Effect of Methylphenidate on Resting-State Connectivity in Adolescents With a Disruptive Behavior Disorder: A Double-Blind Randomized Placebo-Controlled fMRI Study. *Front Psychiatry.* 2021;12:662652.
82. Passamonti L, Fairchild G, Goodyer IM, et al. Neural abnormalities in early-onset and adolescence-onset conduct disorder. *Arch Gen Psychiatry.* 2010;67(7):729-738.
83. Pawliczek CM, Derntl B, Kellermann T, Gur RC, Schneider F, Habel U. Anger under control: neural correlates of frustration as a function of trait aggression. *PLoS One.* 2013;8(10):e78503.
84. Pawliczek CM, Derntl B, Kellermann T, Kohn N, Gur RC, Habel U. Inhibitory control and trait aggression: neural and behavioral insights using the emotional stop signal task. *Neuroimage.* 2013;79:264-274.
85. Perino MT, Guassi Moreira JF, Telzer EH. Links between adolescent bullying and neural activation to viewing social exclusion. *Cogn Affect Behav Neurosci.* 2019;19(6):1467-1478.
86. Philippi CL, Pujara MS, Motzkin JC, Newman J, Kiehl KA, Koenigs M. Altered resting-state functional connectivity in cortical networks in psychopathy. *J Neurosci.* 2015;35(15):6068-6078.
87. Ponseti J, Bruhn D, Nolting J, et al. Decoding Pedophilia: Increased Anterior Insula Response to Infant Animal Pictures. *Front Hum Neurosci.* 2017;11:645.
88. Prehn K, Schulze L, Rossmann S, et al. Effects of emotional stimuli on working memory processes in male criminal offenders with borderline and antisocial personality disorder. *World J Biol Psychiatry.* 2013;14(1):71-78.
89. Pujol J, Batalla I, Contreras-Rodríguez O, et al. Breakdown in the brain network subserving moral judgment in criminal psychopathy. *Soc Cogn Affect Neurosci.* 2012;7(8):917-923.
90. Qi J, Lulu H, Jiang Q, Changran L, Huanzhen W. The relationship between the caudate nucleus-orbitomedial prefrontal cortex connectivity and reactive aggression: A resting-state fMRI study. *Acta Psychologica Sinica.* 2018;50(6):655.
91. Raschle NM, Fehlbaum LV, Menks WM, et al. Atypical Dorsolateral Prefrontal Activity in Female Adolescents With Conduct Disorder During Effortful Emotion Regulation. *Biol Psychiatry Cogn Neurosci Neuroimaging.* 2019;4(11):984-994.
92. Repple J, Habel U, Wagels L, Pawliczek CM, Schneider F, Kohn N. Sex differences in the neural correlates of aggression. *Brain Struct Funct.* 2018;223(9):4115-4124.
93. Rilling JK, Glenn AL, Jairam MR, et al. Neural correlates of social cooperation and non-cooperation as a function of psychopathy. *Biol Psychiatry.* 2007;61(11):1260-1271.

94. Ristow I, Foell J, Kärgel C, et al. Expectation of sexual images of adults and children elicits differential dorsal anterior cingulate cortex activation in pedophilic sexual offenders and healthy controls. *Neuroimage Clin.* 2019;23:101863.
95. Rodman AM, Kastman E, Dorfman HM, et al. Selective Mapping of Psychopathy and Externalizing to Dissociable Circuits for Inhibitory Self-Control. *Clin Psychol Sci.* 2016;4(3):559-571.
96. Rodriguez-Thompson AM, Meyer KM, Davidow JY, et al. Examining cognitive control and reward interactions in adolescent externalizing symptoms. *Dev Cogn Neurosci.* 2020;45:100813.
97. Rubia K, Halari R, Cubillo A, Mohammad AM, Scott S, Brammer M. Disorder-specific inferior prefrontal hypofunction in boys with pure attention-deficit/hyperactivity disorder compared to boys with pure conduct disorder during cognitive flexibility. *Hum Brain Mapp.* 2010;31(12):1823-1833.
98. Rubia K, Halari R, Smith AB, Mohammad M, Scott S, Brammer MJ. Shared and disorder-specific prefrontal abnormalities in boys with pure attention-deficit/hyperactivity disorder compared to boys with pure CD during interference inhibition and attention allocation. *J Child Psychol Psychiatry.* 2009;50(6):669-678.
99. Rubia K, Halari R, Smith AB, et al. Dissociated functional brain abnormalities of inhibition in boys with pure conduct disorder and in boys with pure attention deficit hyperactivity disorder. *Am J Psychiatry.* 2008;165(7):889-897.
100. Rubia K, Smith AB, Halari R, et al. Disorder-specific dissociation of orbitofrontal dysfunction in boys with pure conduct disorder during reward and ventrolateral prefrontal dysfunction in boys with pure ADHD during sustained attention. *Am J Psychiatry.* 2009;166(1):83-94.
101. Sadeh N, Spielberg JM, Heller W, et al. Emotion disrupts neural activity during selective attention in psychopathy. *Soc Cogn Affect Neurosci.* 2013;8(3):235-246.
102. Sakai JT, Dalwani MS, Mikulich-Gilbertson SK, et al. Imaging decision about whether to benefit self by harming others: Adolescents with conduct and substance problems, with or without callous-unemotionality, or developing typically. *Psychiatry Res Neuroimaging.* 2017;263:103-112.
103. Schiffer B, Pawliczek C, Mu Ller B, et al. Neural mechanisms underlying cognitive control of men with lifelong antisocial behavior. *Psychiatry Res.* 2014;222(1-2):43-51.
104. Schiffer B, Pawliczek C, Müller BW, et al. Neural Mechanisms Underlying Affective Theory of Mind in Violent Antisocial Personality Disorder and/or Schizophrenia. *Schizophr Bull.* 2017;43(6):1229-1239.
105. Schultz DH, Balderston NL, Baskin-Sommers AR, Larson CL, Helmstetter FJ. Psychopaths Show Enhanced Amygdala Activation during Fear Conditioning. *Front Psychol.* 2016;7:348.
106. Schwenck C, Ciaramidaro A, Selivanova M, Tournay J, Freitag CM, Siniatchkin M. Neural correlates of affective empathy and reinforcement learning in boys with conduct problems: fMRI evidence from a gambling task. *Behav Brain Res.* 2017;320:75-84.
107. Seara-Cardoso A, Viding E, Lickley RA, Sebastian CL. Neural responses to others' pain vary with psychopathic traits in healthy adult males. *Cogn Affect Behav Neurosci.* 2015;15(3):578-588.

108. Sebastian CL, McCrory EJ, Dadds MR, et al. Neural responses to fearful eyes in children with conduct problems and varying levels of callous-unemotional traits. *Psychol Med.* 2014;44(1):99-109.
109. Sebastian CL, Stafford J, McCrory EJ, et al. Modulation of Amygdala Response by Cognitive Conflict in Adolescents with Conduct Problems and Varying Levels of CU Traits. *Res Child Adolesc Psychopathol.* 2021;49(8):1043-1054.
110. Sethi A, McCrory E, Puetz V, et al. Primary and Secondary Variants of Psychopathy in a Volunteer Sample Are Associated With Different Neurocognitive Mechanisms. *Biol Psychiatry Cogn Neurosci Neuroimaging.* 2018;3(12):1013-1021.
111. Sethi A, O'Nions E, McCrory E, Bird G, Viding E. An fMRI investigation of empathic processing in boys with conduct problems and varying levels of callous-unemotional traits. *Neuroimage Clin.* 2018;18:298-304.
112. Shane MS, Groat LL. Capacity for upregulation of emotional processing in psychopathy: all you have to do is ask. *Soc Cogn Affect Neurosci.* 2018;13(11):1163-1176.
113. Shannon KE, Sauder C, Beauchaine TP, Gatzke-Kopp LM. Disrupted effective connectivity between the medial frontal cortex and the caudate in adolescent boys with externalizing behavior disorders. *Criminal Justice and Behavior.* 2009;36(11):1141-1157.
114. Shao R, Lee TMC. Are individuals with higher psychopathic traits better learners at lying? Behavioural and neural evidence. *Transl Psychiatry.* 2017;7(7):e1175.
115. Siep N, Tonnaer F, van de Ven V, Arntz A, Raine A, Cima M. Anger provocation increases limbic and decreases medial prefrontal cortex connectivity with the left amygdala in reactive aggressive violent offenders. *Brain Imaging Behav.* 2019;13(5):1311-1323.
116. Sommer M, Sodian B, Döhnel K, Schwerdtner J, Meinhardt J, Hajak G. In psychopathic patients emotion attribution modulates activity in outcome-related brain areas. *Psychiatry Res.* 2010;182(2):88-95.
117. Sterzer P, Stadler C, Krebs A, Kleinschmidt A, Poustka F. Abnormal neural responses to emotional visual stimuli in adolescents with conduct disorder. *Biol Psychiatry.* 2005;57(1):7-15.
118. Szczypiński J, Wypych M, Krasowska A, et al. Abnormal behavioral and neural responses in the right dorsolateral prefrontal cortex during emotional interference for cognitive control in pedophilic sex offenders. *J Psychiatr Res.* 2022;151:131-135.
119. Taubner S, Hauschild S, Wisniewski D, Wolter S, Roth G, Fehr T. Neural response to aggressive and positive interactions in violent offenders and nonviolent individuals. *Brain Behav.* 2021;11(12):e32400.
120. Thornton LC, Penner EA, Nolan ZT, et al. The processing of animacy information is disrupted as a function of callous-unemotional traits in youth with disruptive behavior disorders. *Neuroimage Clin.* 2017;16:498-506.
121. Uytun MC, Karakaya E, Oztop DB, et al. Default mode network activity and neuropsychological profile in male children and adolescents with attention deficit hyperactivity disorder and conduct disorder. *Brain Imaging Behav.* 2017;11(6):1561-1570.
122. van Hoorn J, McCormick EM, Perino MT, Rogers CR, Telzer EH. Differential Behavioral and Neural Profiles in Youth With Conduct Problems During Risky Decision-Making. *J Res Adolesc.* 2020;30(3):599-615.

123. Vanova M, Aldridge-Waddon L, Norbury R, Jennings B, Puzzo I, Kumari V. Distinct neural signatures of schizotypy and psychopathy during visual word-nonword recognition. *Hum Brain Mapp.* 2022.
124. Varkevisser T, Gladwin TE, Heesink L, van Honk J, Geuze E. Resting-state functional connectivity in combat veterans suffering from impulsive aggression. *Soc Cogn Affect Neurosci.* 2017;12(12):1881-1889.
125. Viding E, Sebastian CL, Dadds MR, et al. Amygdala response to preattentive masked fear in children with conduct problems: the role of callous-unemotional traits. *Am J Psychiatry.* 2012;169(10):1109-1116.
126. Völlm B, Richardson P, McKie S, Elliott R, Dolan M, Deakin B. Neuronal correlates of reward and loss in Cluster B personality disorders: a functional magnetic resonance imaging study. *Psychiatry Res.* 2007;156(2):151-167.
127. Völlm B, Richardson P, McKie S, et al. Neuronal correlates and serotonergic modulation of behavioural inhibition and reward in healthy and antisocial individuals. *J Psychiatr Res.* 2010;44(3):123-131.
128. Werhahn JE, Mohl S, Willinger D, et al. Aggression subtypes relate to distinct resting state functional connectivity in children and adolescents with disruptive behavior. *Eur Child Adolesc Psychiatry.* 2021;30(8):1237-1249.
129. White SF, Fowler KA, Sinclair S, et al. Disrupted expected value signaling in youth with disruptive behavior disorders to environmental reinforcers. *J Am Acad Child Adolesc Psychiatry.* 2014;53(5):579-588.e579.
130. White SF, Marsh AA, Fowler KA, et al. Reduced amygdala response in youths with disruptive behavior disorders and psychopathic traits: decreased emotional response versus increased top-down attention to nonemotional features. *Am J Psychiatry.* 2012;169(7):750-758.
131. White SF, Pope K, Sinclair S, et al. Disrupted expected value and prediction error signaling in youths with disruptive behavior disorders during a passive avoidance task. *Am J Psychiatry.* 2013;170(3):315-323.
132. White SF, Thornton LC, Leshin J, et al. Looming Threats and Animacy: Reduced Responsiveness in Youth with Disrupted Behavior Disorders. *J Abnorm Child Psychol.* 2018;46(4):741-754.
133. White SF, VanTieghem M, Brislin SJ, et al. Neural Correlates of the Propensity for Retaliatory Behavior in Youths With Disruptive Behavior Disorders. *Am J Psychiatry.* 2016;173(3):282-290.
134. White SF, Williams WC, Brislin SJ, et al. Reduced activity within the dorsal endogenous orienting of attention network to fearful expressions in youth with disruptive behavior disorders and psychopathic traits. *Dev Psychopathol.* 2012;24(3):1105-1116.
135. Yoder KJ, Harenski C, Kiehl KA, Decety J. Neural networks underlying implicit and explicit moral evaluations in psychopathy. *Transl Psychiatry.* 2015;5(8):e625.
136. Yoder KJ, Harenski C, Kiehl KA, Decety J. Neural responses to morally laden interactions in female inmates with psychopathy. *Neuroimage Clin.* 2021;30:102645.
137. Yoder KJ, Harenski CL, Kiehl KA, Decety J. Psychopathic traits modulate functional connectivity during pain perception and perspective-taking in female inmates. *Neuroimage Clin.* 2022;34:102984.

138. Yoder KJ, Porges EC, Decety J. Amygdala subnuclei connectivity in response to violence reveals unique influences of individual differences in psychopathic traits in a nonforensic sample. *Hum Brain Mapp.* 2015;36(4):1417-1428.
139. Zhang J, Li B, Gao J, et al. Impaired Frontal-Basal Ganglia Connectivity in Male Adolescents with Conduct Disorder. *PLoS One.* 2015;10(12):e0145011.
140. Zhu W, Zhou X, Xia LX. Brain structures and functional connectivity associated with individual differences in trait proactive aggression. *Sci Rep.* 2019;9(1):7731.
141. Zhu Y, Ying K, Wang J, et al. Differences in functional activity between boys with pure oppositional defiant disorder and controls during a response inhibition task: a preliminary study. *Brain Imaging Behav.* 2014;8(4):588-597.
